# AI discovery of TLR agonist-driven phenotypes reveals unique features of peripheral cells from healthy donors and ART-suppressed people living with HIV

**DOI:** 10.3389/fimmu.2025.1541152

**Published:** 2025-03-25

**Authors:** Robert Were Omange, Samuel C. Kim, Nikita S. Kolhatkar, Tempest Plott, Will Van Trump, Kenneth Zhang, Hope O’Donnell, Daniel Chen, Ahmed Hosny, Michael Wiest, Zach Barry, Elisa Cambronero Addiego, Meron Mengistu, Pamela M. Odorizzi, Yanhui Cai, Rachel Jacobson, Jeffrey J. Wallin

**Affiliations:** ^1^ Biomarker Sciences and Diagnostics, Gilead Sciences, Inc., Foster City, CA, United States; ^2^ Spring Science, San Carlos, CA, United States

**Keywords:** TLR, HIV, AI, ML, high content imaging

## Abstract

**Background:**

Selective and potent Toll-like receptor (TLR) agonists are currently under evaluation in preclinical models and clinical studies to understand how the innate immune system can be harnessed for therapeutic potential. These molecules are designed to modulate innate and adaptive immune responses, making them promising therapeutic candidates for treating diseases such as cancer or chronic viral infections. Much is known about the expression and signaling of TLRs which varies based on cell type, cellular localization, and tissue distribution. However, the downstream effects of different TLR agonists on cellular populations and phenotypes are not well understood. This study aimed to investigate the impact of TLR pathway stimulation on peripheral blood mononuclear cell (PBMC) cultures from people living with HIV (PLWH) and healthy donors.

**Methods:**

The effects of TLR4, TLR7, TLR7/8, TLR8 and TLR9 agonists were evaluated on cytokine production, cell population frequencies, and morphological characteristics of PBMC cultures over time. Changes in the proportions of different cell populations in blood and morphological features were assessed using high-content imaging and analyzed using an AI-driven approach.

**Results:**

TLR4 and TLR8 agonists promoted a compositional shift and accumulation of small round (lymphocyte-like) PBMCs, whereas TLR9 agonists led to an accumulation of large round (myeloid-like) PBMCs. A related increase was observed in markers of cell death, most prominently with TLR4 and TLR8 agonists. All TLR agonists were shown to promote some features associated with cellular migration. Furthermore, a comparison of TLR agonist responses in healthy and HIV-positive PBMCs revealed pronounced differences in cytokine/chemokine responses and morphological cellular features. Most notably, higher actin contraction and nuclear fragmentation was observed in response to TLR4, TLR7, TLR7/8 and TLR9 agonists for antiretroviral therapy (ART)-suppressed PLWH versus healthy PBMCs.

**Conclusions:**

These data suggest that machine learning, combined with cell imaging and cytokine quantification, can be used to better understand the cytological and soluble immune responses following treatments with immunomodulatory agents *in vitro.* In addition, comparisons of these responses between disease states are possible with the appropriate patient samples.

## Introduction

The innate immune system has evolved the capacity to recognize molecules with highly conserved pathogen associated molecular patterns (PAMPs) through pattern recognition receptors (PRRs), the most prominent being the Toll-like receptor (TLR) family (1). These interactions ultimately play a critical role in the development of adaptive immunity against infectious diseases (2, 3). TLR expression varies based on immune cell type, and the downstream effects of TLR-mediated immune activation include the induction of cytokines and chemokines, antimicrobial proteins, and upregulation of costimulatory molecules on antigen presenting cells, and activate cells associated with innate and adaptive immune responses (4). The TLR-mediated effects on cellular activation are fundamental to both the quality and magnitude of innate immune responses, influencing diverse cellular functions and immune outcomes. The ability to elicit both innate and adaptive immune responses makes TLRs attractive targets for therapeutic interventions (5). Synthetic TLR agonists that can selectively target their cognate receptors have been developed to boost immune responses against infections or malignancies (6, 7). Emerging strategies include the use of TLR agonists as part of combination therapeutic strategy with other treatment modalities, such as vaccines or broadly neutralizing antibodies (8–11).

The expression and functions of TLRs are altered during the acute, chronic, or acquired immunodeficiency stages of human immunodeficiency virus (HIV) disease (2). Recognition of TLR7 and TLR8 viral PAMPs—single-stranded RNA—plays a pivotal role in sustaining and inducing the deleterious immune activation which drives HIV pathogenesis (12, 13). HIV infection dysregulates TLR expression and functions, which are partially restored by antiretroviral therapy (ART) (14, 15). Many of the studies conducted to characterize the expression and functions of different TLRs during HIV infections have focused on immunological and signaling mechanisms (16–18). However, it remains poorly understood how different TLRs impact cellular morphology and complex immunological functional readouts in HIV-infected versus healthy PBMCs.

Here, we used machine learning to extract and analyze morphological embeddings from cell culture images of human peripheral blood mononuclear cells (PBMCs) from healthy volunteers (HV) and ART-suppressed people living with HIV (PLWH) stimulated with various TLR agonists. The selected TLRs included endosomal TLR7, TLR8 and TLR9, and surface TLR4, where TLR7 and TLR8 are also known to play a critical role in HIV pathogenesis and have well characterized cytokine profiles in both healthy subjects and in the context of HIV infection (19–21). Images obtained from TLR stimulated PBMCs of HV and PLWH, were used to train supervised and unsupervised models that differentiated the downstream effects of the TLR pathways signaling on cellular morphology and immune mediated functions. The method involved a multi-faceted computational approach that identified and distinguished unique cellular states associated with the specific TLR pathway stimulation. The interpretable biological metrics uncovered similarities between TLR4 and TLR8 agonists, which were distinct from TLR7 and TLR9 agonist responses.

## Materials and methods

### Study design and samples

Cryopreserved PBMC samples collected from 30 healthy volunteers (HV) and 10 antiretroviral therapy (ART)-suppressed PLWH were used in this study. PBMCs from HV were obtained under the Gilead blood collection program, while PBMCs collected from ART suppressed PLWH were procured by Cell Quest under the protocol “Leukapheresis on Adult Participants with HIV-1 to Obtain Leukocytes for Testing of Virus Eradication Strategies Protocol”. PLWH were selected based on the following inclusion criteria: Either male or female, at least 18 years of age, and diagnosed with HIV-1 infection. All participants had to be on an ART regimen for at least 12 months prior to enrollment in the study and had undetectable plasma viral loads below 50 copies per mL based on Cobas (Roche) Amplicor (or similar) assay. Additionally, all participants had well preserved CD4 cell counts above 350 cells/ml. Individuals who tested positive for hepatitis B or C viruses or had a history of ART drug-resistance, switched regimens, or women who were or became pregnant or breastfeeding, were excluded from the study. All study participants provided informed consent prior to enrollment into the study.

### TLR stimulation

Cryopreserved PBMCs were thawed in a 37°C water bath, washed in standard media and resuspended in PBMC plating media (RPMI with 10% FBS and supplements) to bring the final cell count per well to 35,000, as calculated from the pre-thaw PBMC count. A Microlab STAR liquid handler (Hamilton) was used to add 20 μL of the cell’s suspension into each well of 384-well plates (Greiner), pre-filled with 10 μL PBMC plating media at room temperature. The numbers of PBMCs in each well were very consistent (**Supplementary Figure S1A**). The plate was incubated at room temperature for 10 minutes, then centrifuged for one minute at 140 RCF, followed by an incubation at 37°C and 5% CO_2_ for 30 minutes to promote attachment to the plate. FBS was then added to bring the final concentration to 10% per well prior to addition of the different TLR stimulants. Two concentrations of each TLR agonists were evaluated, either high or low, except TLR4 (**Table 1**). The appropriate concentration of TLR agonists was then added into each well of a 384 well plate, using a Mantis liquid dispenser (Formulatrix), and incubated for 6, 24, or 48 hours at 37°C, 5% CO_2_.

**Table 1:** Concentrations of TLR agonists used in this study.

TLR Receptor	Compound	Assay Concentration
TLR4	Lipopolysaccharide (LPS) (Invivogen)	0.1 µg/mL
TLR7	Vesatolimod (Gilead)	0.1 or 1.0 µM
	Imiquimod (Invivogen)	1.0 or 10.0 µM
TLR7/8	Resiquimod (Invivogen)	0.025 or 0.25 µg/mL
TLR8	Motolimod (MCE)	0.1 or 5.0 µM
	Selgantolimod (MCE)	0.1 or 1.1 µM
TLR9	CpG 2395 (Invivogen)	1.0 or 10.0 µM
	CpG 2006 (Invivogen)	1.0 or 10.0 µM
Control	DMSO	0.2% v/v

At the end of each incubation period, cell culture supernatants were collected from each well and stored at −80°C. These were later thawed, treated with 1% Triton X-100 for 2 hours at room temperature, and refrozen at −80°C before shipping on dry ice to Nomic Bio (Montreal) for the quantification of cytokine and chemokine responses.

### Secreted protein analysis

Frozen cell-culture supernatants were shipped on dry ice to Nomic Bio (Montreal) for analysis of a custom panel of 51 cytokines and chemokines using standard protocols (22). Briefly, the frozen supernatants were thawed and diluted 1:1 using R10 media (RPMI 1640 supplemented with 10% heat inactivated FBS, 2% Pen/Strep), and nELISA beads were added to each well of the 384 well plates and incubated for 3 hours at room temperature. The target-bound beads were washed with Wash Buffer and resuspended in Assay Buffer, prior to the addition of Displacement Oligo, all reagents included in the assay kit. The target-bound-oligo-bead mixture was then incubated at room temperature for 30 minutes, then washed with Wash Buffer and resuspended in Assay Buffer. The target-bound-oligo beads were then read by flow cytometry on a ZE5 Cell Analyzer (Bio-Rad).

Secreted protein data was normalized as fold-changes above the reference DMSO/basal control per plate, donor, and protein grouping. The median concentrations for the controls and analytes (serum protein-cytokine, chemokine or factor), were calculated. The fold-change per analyte per donor was averaged to generate per-donor measurements, and per donor measurements compared between groups.

### Immunofluorescence staining for imaging

PBMCs were stained with 0.5 µM MitoTracker Deep Red FM (ThermoFisher) and 0.5 µg/mL Annexin V CF750 (Biotium) for 30 minutes at 37°C and 5% CO^2^. The stained cells were then fixed with 4% paraformaldehyde (PFA) for 15 minutes at room temperature, blocked, and permeabilized using 1% bovine growth serum and 0.3% Triton X-100 in phosphate-buffered saline (PBS) for 15 minutes at room temperature. Following fixation and permeabilization, the cells were stained with Concanavalin A Alexa Fluor 488 (ThermoFisher) at 25 µg/mL and Phalloidin Phenovue Fluor 568 (Perkin Elmer) at 66 nM in staining buffer (1% bovine growth serum in PBS) overnight at 4°C. Finally, the cells were washed 3 times with PBS, stained with Hoechst 33342 (ThermoFisher) at 0.2 µg/mL in PBS for 15 min, and finally washed 3× times with PBS, prior to imaging. The fixation, washing, and staining steps were done on a Biotek EL406 washer dispenser (Agilent). Imaging was performed on an ImageXpress^®^ Micro Confocal high content imager (Molecular Devices).

### Computational image analysis

Extracting image features using artificial intelligence embeddings was performed as previously described (23). High content images were captured for the entire contents of each well, and each image was scored for an ‘on basis’ and ‘off basis’ effect (24). ‘On basis’ referring to the distance along the projected vector in an embedded space from the centroid of the negative control cluster to the centroid of the positive control cluster. ‘Off basis’ was calculated as the orthogonal distance from projection to corresponding data points per embedded space. While assay performance metrics for phenotypic assays rarely achieved levels of significance seen in target- or molecular-based assays, for this study we achieved a beta value (strictly standardized mean difference) of 2.68, which is interpreted as excellent or good strengths of differences for moderate or strong positive controls, respectively (25) (**Supplementary Figure S1B**).

#### Image processing and morphological feature extraction

Fluorescence images were trained to identify cells and produce phenotypic embedding representations of each cell. Individual cells were first identified via nuclei channel segmentation with the CellProfiler pipeline (26) and the deep learning-based StarDist algorithm (27–29). Following identification, cell-sized image crops were centered on the centroid of each cell nucleus, and embeddings produced for each crop. The images were fed into the RepLKNet (30) architecture backbone (with a global average pool of the last convolutional layer followed by a 1D average pool to generate a 128-long embedding vector). RepLKNet is a network trained on a multitude of real-world/photographic images from the ImageNet-21K dataset (31). Each embedding produced comprised of an abstract “black box” representation of morphological features such as cell shape and texture that are used in this work as an alternative to more traditional, image-processing-based features such as area, eccentricity, among others, used for downstream applications such as single-cell classifiers to sort cells into predefined phenotypic bins. In total, approximately 410,000 images (24 plates × 384 wells × 9 field of views × 5 fluorescence channels) with 2,048 × 2,048 pixels were processed for feature extraction. A more detailed description of computational methods can be found in (23).

Quality control processes were optimized to evaluate high-content imaging to ensure both data consistency and high-quality images for machine learning (ML) analyses. These previously established parameters (32, 33) included (a) cell count consistency, (b) cell composition consistency across donors, plates, and time points, (c) Consistency of cell staining and (d) Imaging focal consistency.

#### Single-cell classifiers (PhenoSorter)

After segmentation and generation of the embeddings, cell phenotypes of interest are identified and training sets of hand-labeled examples generated using the PhenoSorter tool in the Spring Science analytical software application. PhenoSorter allows users to manually label phenotypes of cells and provide examples of defined classes visually (34). Separate sets and corresponding PhenoSorter models were created for phenotypic categories of interest. Models were initially trained with a k-nearest neighbors’ approach, using 80/20 random split of cell samples for training/testing and evaluated for adequate model performance for each model class using example cell crops held out from the training procedure. Balanced class weighting was used such that the model is penalized more for misclassifying classes with fewer samples. Once a desired performance per class was achieved for each model, a final XGBoost-based model was trained and the resulting model applied to the entire dataset used to sorting the cells into the class bins for the model. The percentage of cells that fall into each bin was then reported.

### TLR expression in public RNA seq datasets

TLR messenger RNA transcript expression calculated as nTPM (normalized transcripts per million), from whole blood of 6 healthy donors sorted by flow cytometry into 18 immune cell types, was retrieved from Human Protein Atlas database (35). Single-cell RNA sequencing (scRNAseq) datasets generated from PBMCs of healthy donors were retrieved from 10x Genomics database for PLWH and NCBI SRA BioProject PRJNA783363 (36) (132,247 cells in total). Analysis of transcriptomic data was performed using Seurat R package (version 5.1) (37, 38). The gene counts were normalized and scaled using the following quality control filters: Cells with less than 200 unique genes detected or with greater than 5% mitochondrial genes were excluded from the analysis. 2,000 variable genes were identified, and 30 principal components (PC) used for dimensionality reduction. Shared Nearest Neighbor (SNN) graph (K = 20) and Louvain method were used for cell type clustering with the resolution parameter of 0.5. Uniform Manifold Approximation and Projection (UMAP) embeddings were calculated for data visualization using the first 30 PC dimensions. The datasets were integrated using Harmony R package (version 1.2) (39).

### Statistical analysis

Statistical analysis used are defined in the figure legends.

## Results

### Experimental approach

**Figure 1:**
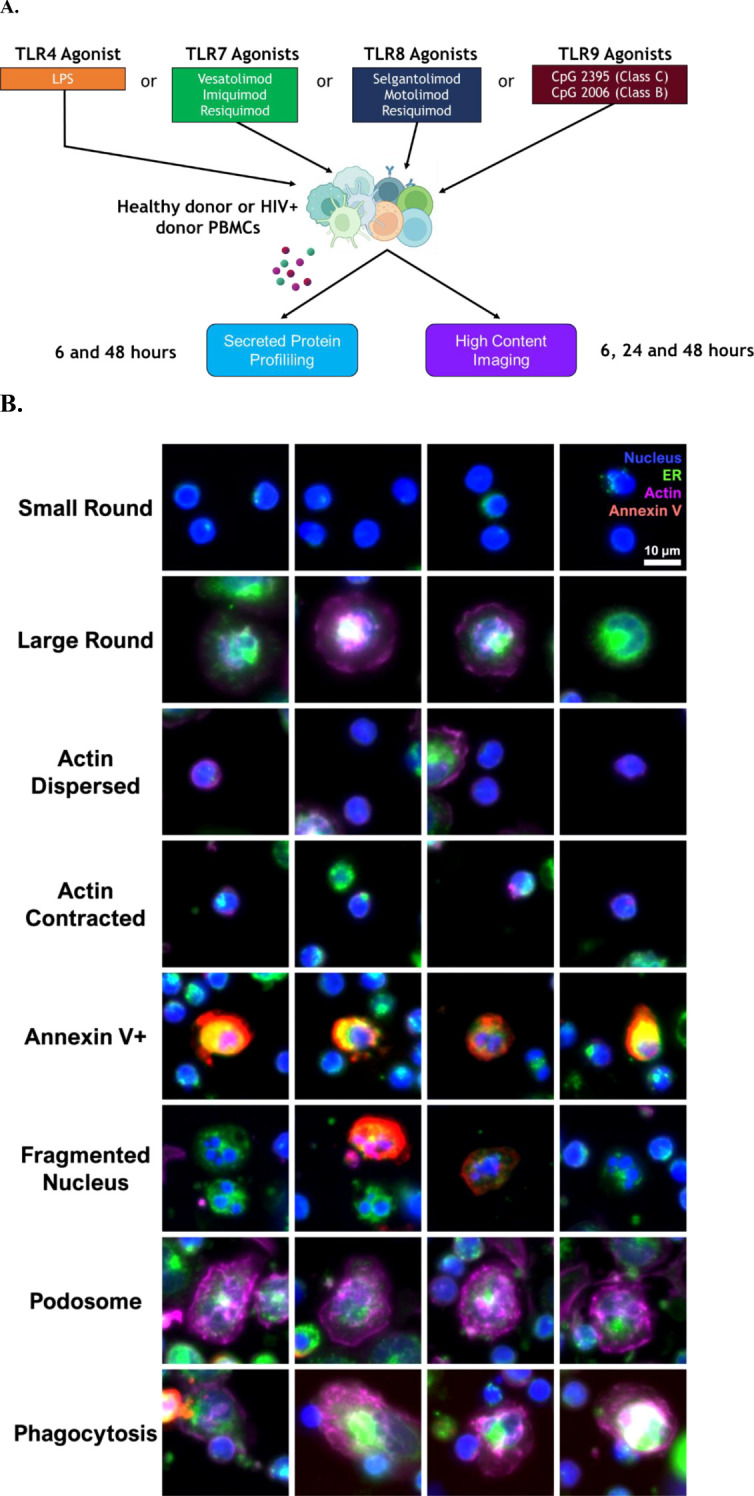
*In vitro* profiling of TLR agonist stimulation in PBMC cultures. **(A)** Experimental approach to analyze secreted proteins and cellular features. **(B)** Examples of cellular features quantified using high content imaging of cultures. Staining to identify cell nucleus (Hoechst, blue), actin (phalloidin, purple), cell membrane/endoplasmic reticulum (concanavalin A, green), and dead/dying cells (annexin V, red).

A selection of TLR agonists were profiled *in vitro* using human PBMCs from healthy donors and ART-suppressed PLWH (**Figure 1A**, **Table 1**). TLR agonist concentrations used and summarized in **Table 1**, were previously validated to be sufficient to stimulate healthy human PBMCs over 48 hours *in vitro* (data not shown). Using PBMCs from 30 healthy donors across 3 timepoints (6 hours, 24 hours, and 48 hours), quality control experiments were conducted to build robust models of TLR activation for both fluorescence imaging and cytokine profiling (**Supplementary Figure S1**).

### Expression of TLRs in PBMCs from healthy volunteers and PLWH

**Figure 2:**
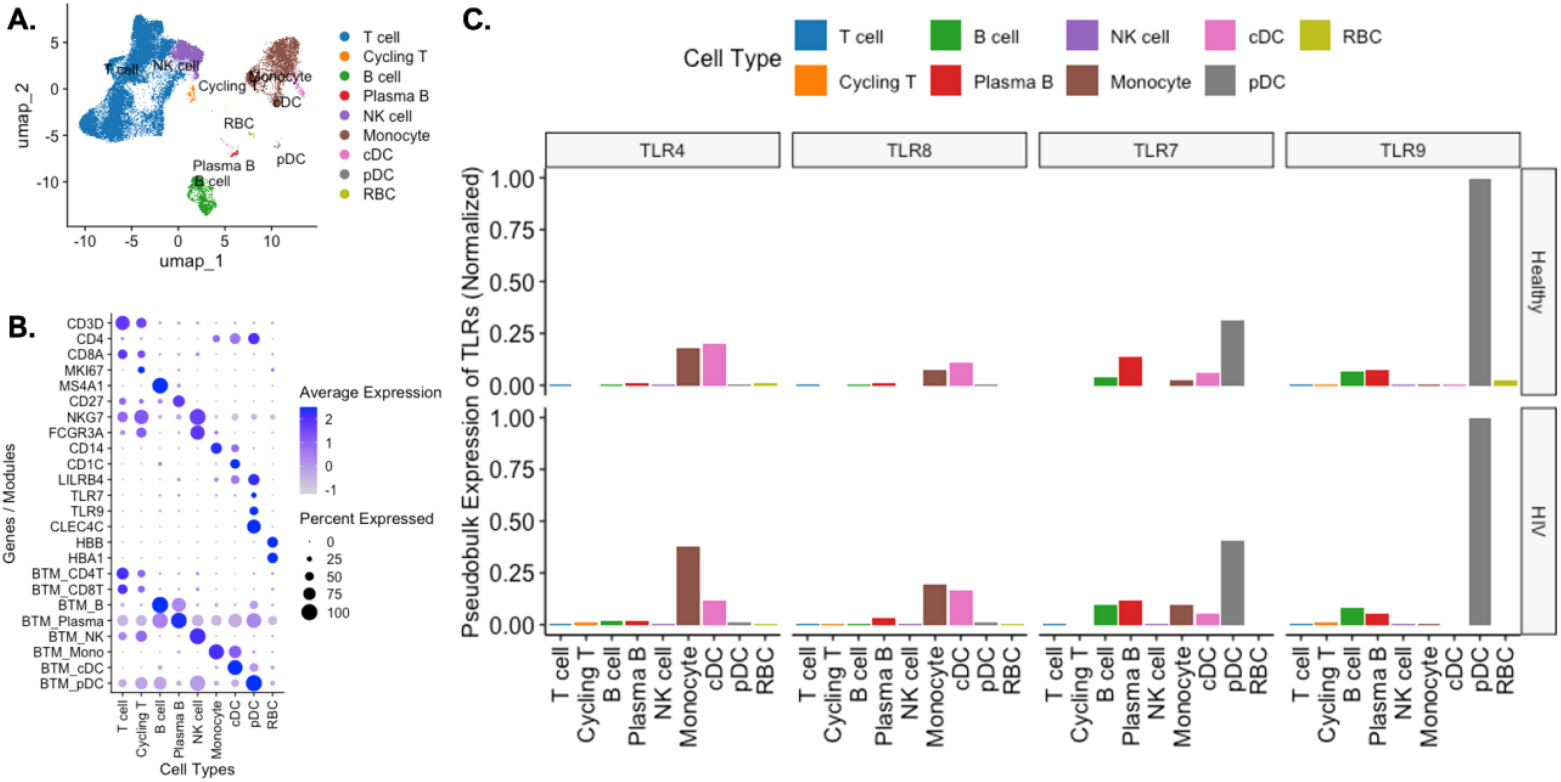
Cellular expression of profiled TLRs in healthy and HIV positive PBMCs from public scRNAseq datasets. **(A)** PBMCs from 2 healthy individuals and 8 PLWH on ART were filtered to exclude low quality cells and doublets and were clustered and displayed in a UMAP plot. **(B)** Cell types were assigned to each cluster using selected marker genes and blood transcription module scores. **(C)** Pseudobulk normalized expression of TLR4, TLR7, TLR8, and TLR9.

To understand TLR mRNA expression patterns in PBMCs, we retrieved publicly available RNAseq datasets and analyzed mRNA gene expression levels of TLR4, TLR7, TLR8 and TLR9 (35, 36). The single cell RNA sequencing (scRNA seq) data from healthy volunteers and PLWH were clustered into nine immune cell types: T cells, cycling T cells, B cells, plasma cells, natural killer (NK) cells, monocytes, conventional dendritic cells (cDCs), plasmacytoid dendritic cells (pDCs) and red blood cells (RBCs) (**Figure 2A**). The immune cell types were assigned based on phenotypic marker gene expression levels and blood transcription module (BTM) scores (**Figure 2B**, **Supplementary Table S1**) (40). Pseudobulk expression levels of TLRs across these immune cells were consistent with the literature (35): TLR4 and TLR8 were primarily expressed on monocytes and cDCs, whereas TLR7 and TLR9 were predominantly expressed on pDCs and B cells (**Figure 2C**). TLR7 was also expressed in monocytes and cDCs, unlike TLR9. These transcriptomic expression patterns were consistent with the bulk RNAseq data from blood cells sorted by flow cytometry (**Supplementary Figure S2**). Within the data from healthy individuals and ART-suppressed PLWH, no significant differences in TLR mRNA distribution over the different immune cell types was observed (**Figure 2C**).

### Levels of many cytokines and chemokines are increased upon TLR stimulation in healthy donor PBMCs

**Figure 3:**
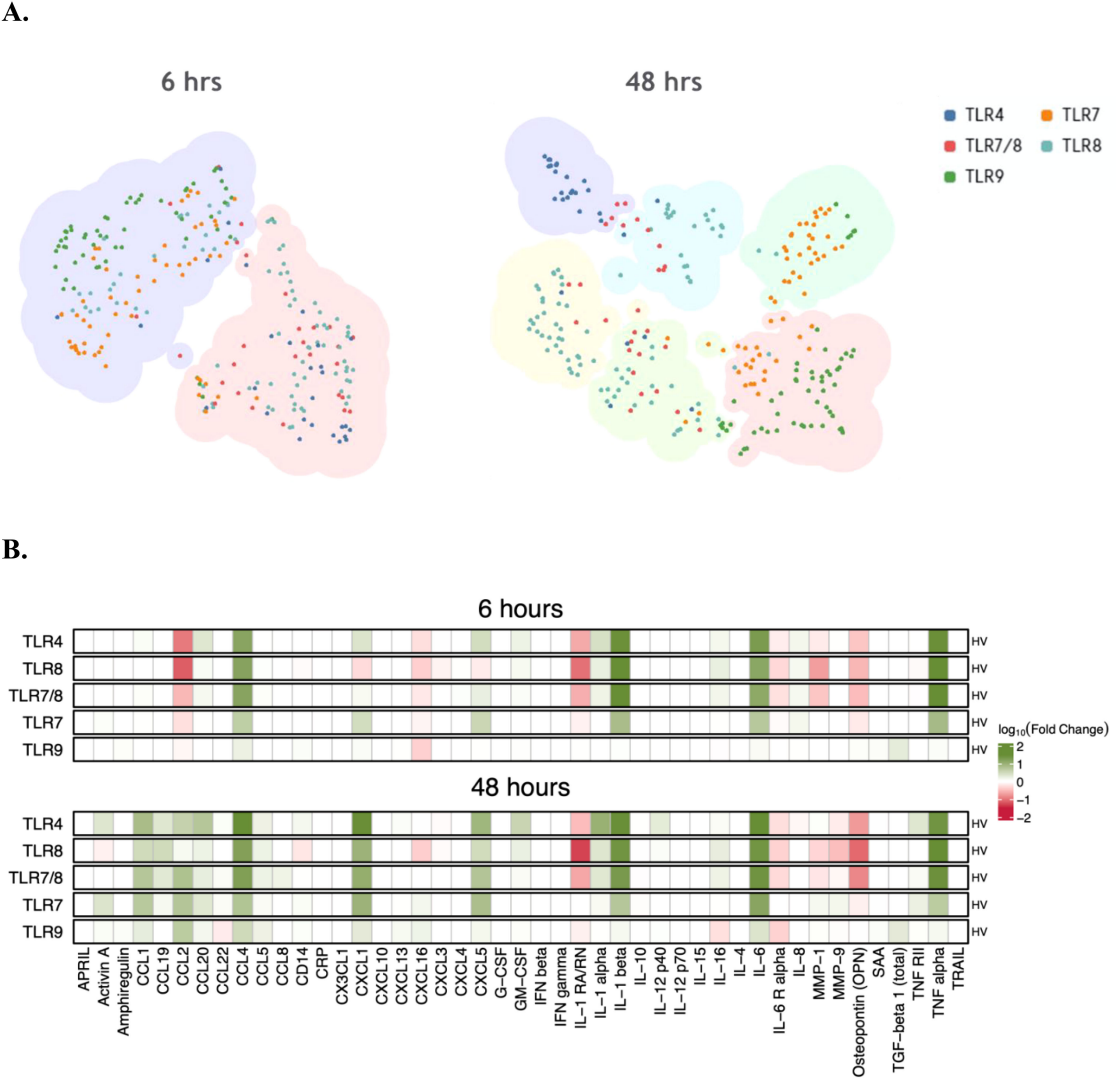
Change in cytokines in healthy PBMC cultures stimulated with TLR agonists. **(A)** TLR stimulation clusters based on changes in cytokine vs DMSO at 6 and 48 hrs of treatment in culture. Each larger cluster color indicates similarity and overlap in cytokine profile at that timepoint **(B)** Changes of specific cytokines in response to specific TLR agonist treatment at 6 and 48 hrs of treatment.

To characterize the specific cytokine, chemokine and soluble analyte profiles elicited by each TLR pathway in healthy volunteers (HV), supernatants were collected 6- and 48-hours post-stimulation with TLR4, TLR7, TLR7/8, TLR8 and TLR9 agonists. Supernatants were evaluated using a cytokine and chemokine multiplex assay, and the magnitude of fold change in cytokine responses upon TLR stimulation was normalized to those responses upon DMSO stimulation for each donor. At 6 hours post-stimulation, cytokine and chemokine shifts were observed in wells stimulated by TLR4, TLR7/8, and TLR8 agonists. Specifically, there were greater fold increases in CCL4, IL-1β, IL-6, and TNF-α and fold decreases in CCL2, IL-1Rα, MMP-1, OPN, and IL-6Rα (**Figure 3B**). TLR7-associated stimuli resulted in similar increases as TLR4 and TLR8 (**Figure 3A**). However, increases in CXCL1 and CXCL5 were also observed while PBMCs stimulated with TLR9 agonists had minimal fluctuations in cytokine profiles at 6 hours.

At 48 hours post-stimulation, increases in CCL1, CCL2, CCL4, CXCL1 and IL-6 were observed in all wells with all TLR stimuli to varying degrees of magnitude fold change. Like the 6-hour timepoint post-stimulation, the magnitude of cytokine responses was greater in the TLR4, TLR7 and TLR8 pathways relative to TLR9.

### TLR stimulation led to differential effects on small and large round cell populations

Based on shape and size, we investigated if the TLR agonists influenced the proportions of specific PBMC sub-populations in culture. Two subsets were expected, “small round cells” (which range from 6 to 15 µm, to be lymphocytes) and “large round cells” (with an approximate size of 20 µm, to be monocytes or monocyte-derived macrophages). Small round cells had minimal cytoplasm and prominently featured nuclei, as indicated by Hoechst staining. Large round cells, however, were presented with an array of definable features using other cell biological stains (e.g., F-actin for cytoskeleton) in addition to the nucleus given their pronounced cytoplasmic areas. Examples of the described small and large round cells are shown in **Figure 1B**. These populations were quantified longitudinally across healthy PBMC cultures. We limited our identification and quantification to round cells, which excluded the quantification of dendritic cells, as they are expected to have a prevalence of only 1⁠–2% in PBMCs.

**Figure 4:**
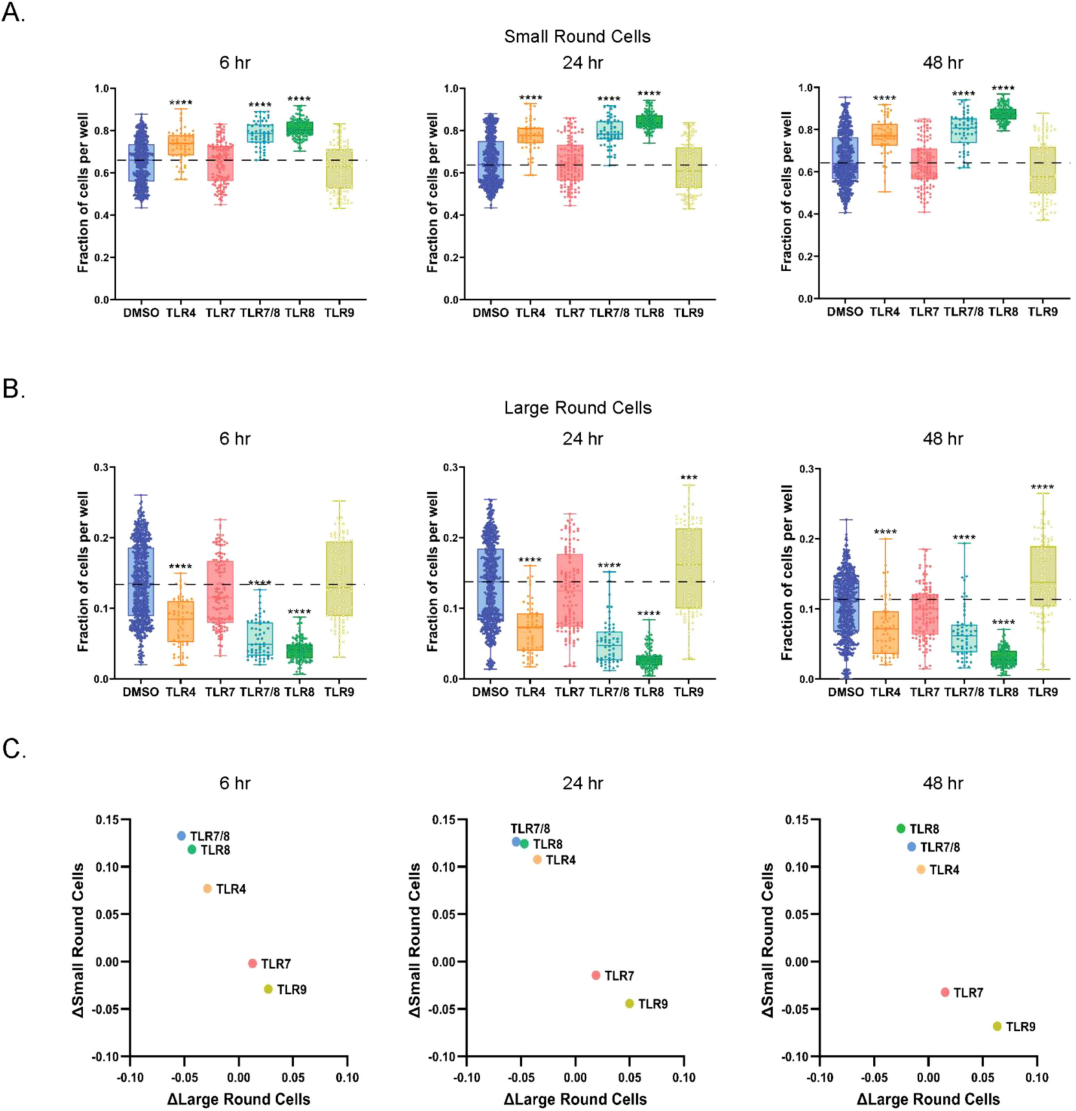
TLR4 and TLR8 agonists promote the accumulation of small round cells. **(A)** Quantification of small round cells at 6, 24, and 48 hrs of culture with DMSO or TLR agonists. Data is shown as a fraction of total small round cells per well. **(B)** Measurements of large round cells at selected timepoints after DMSO or TLR agonist treatment. Data is shown as fraction of total large round cells per well. **(C)** Relative changes to DMSO in fractions of small and large round cells in PBMC cultures at 6, 24, and 48 hrs of culture. Statistical comparison relative to DMSO was done using a Mann-Whitney U test, ****p < 0.0001, ***p < 0.001. The horizontal dotted lines in **(A-C)** represent DMSO average, with the means, minimum and maximum observation indicated by error bar, while the boxes represent the interquartile range.

The fraction of small round cells with TLR4 and TLR8 agonist stimulations was higher than the DMSO control across all three timepoints (**Figure 4A**). This effect was most pronounced with TLR8 agonists and was significantly higher than TLR4 pathway stimulation at all three timepoints. The small round cell frequency was higher with TLR7/8 agonists stimulations than DMSO control, but not those with TLR7 agonists, suggesting the compositional shift towards small round cells was driven primarily by their TLR8 activity. TLR9 agonists did not have a significant effect on small round cell populations across the time course.

In contrast, the fraction of large round cells decreased relative to DMSO in response to TLR4 and TLR8 agonists across all time points (**Figure 4B**). The effect was more noticeable for TLR8 agonists than for TLR4 agonists. Once again, the effects of TLR7/8 agonists paralleled TLR8 pathway activation but remained distinct from TLR7 agonists. We also observed a higher frequency of large round cells in those stimulated with TLR9 pathway agonists than those stimulated with DMSO, but only after 24 hours of culture. We did not detect a difference in small or large round cell populations for those stimulated with TLR7 agonist at any time point. Overall, individual TLR agonists targeting the same receptor showed similar trends to one another (**Supplementary Figures S3A, B**). Correlation analysis for the different TLR mechanisms confirmed that TLR4 and TLR8 pathway stimulations had similar effects on large and small round cell populations, while TLR7 and TLR9 pathway stimulation were more analogous to each other (**Figure 4C**).

### Features of cell death are most prominently increased with TLR8 agonist stimulation

**Figure 5:**
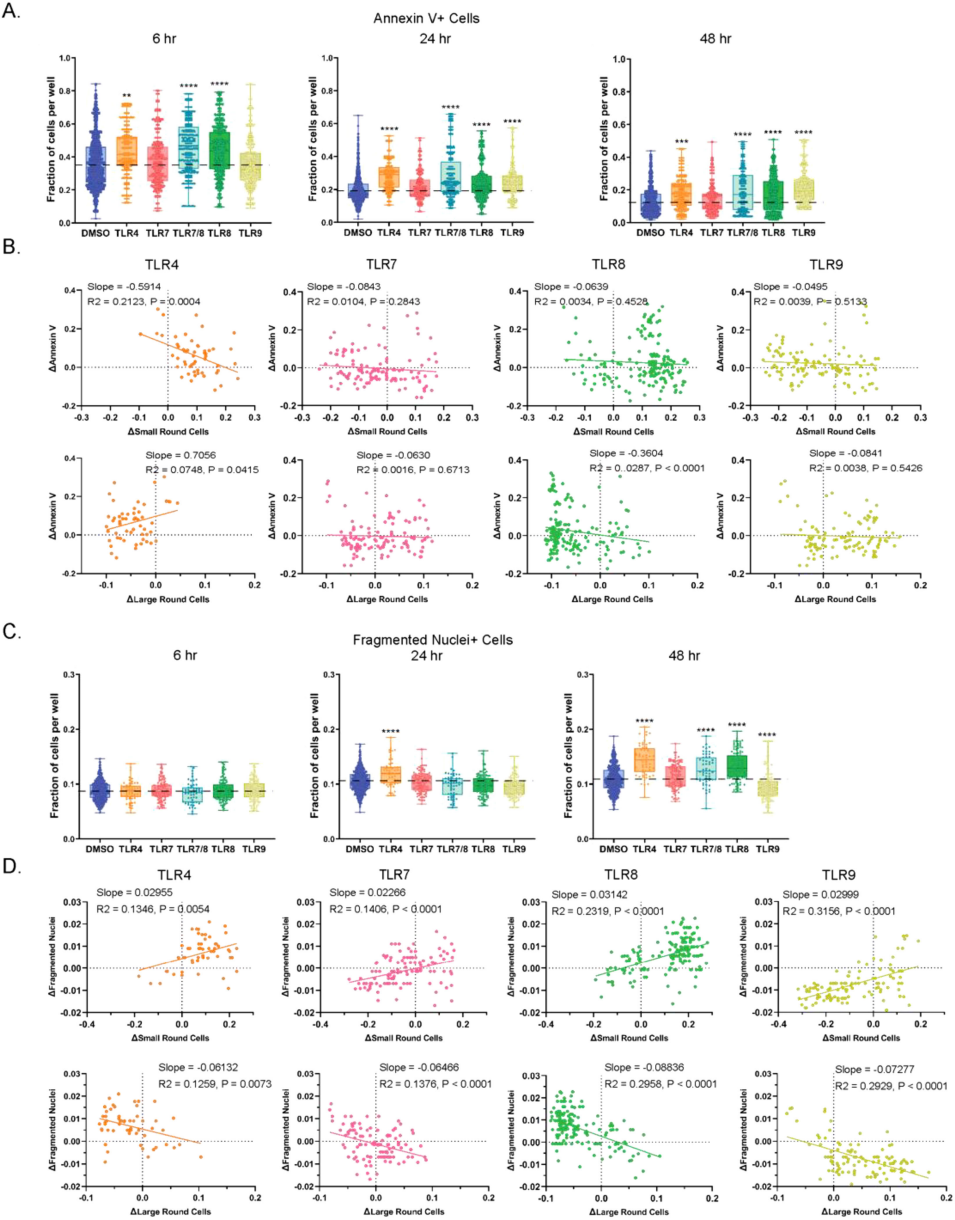
Increases in cell features were observed in cultures stimulated with TLR4 and TLR8 agonists. **(A)** Fraction of annexin V+ cells per well in DMSO control and TLR agonist stimulated cell cultures. Data are shown for 6, 24, and 48 hrs of treatment. **(B)** Changes relative to DMSO at 24 hrs of treatment in annexin V positivity of small and large round cells in cultures treated with TLR agonists. **(C)** Quantification of fragmented nuclei positive cells in DMSO and TLR agonist stimulated cultures at 6, 24, and 48 hr timepoints. **(D)** Changes relative to DMSO in fragmented nuclei positivity of small and large round cells in cultures treated with TLR agonists. Statistical comparison relative to DMSO was done using a Mann-Whitney U test, **** *p* < 0.0001, *** *p* < 0.001, ** *p* < 0.01 and correlational analysis done using Spearman’s correlation with the slopes R^2^ and *p* values indicated. The horizontal dotted lines in A and C represent DMSO average, with the means, minimum and maximum observation indicated by error bar, while the boxes represent the interquartile range.

To determine if the TLR-induced changes in proportions of small and large round cell populations may be due to cell death, cell surface expression of annexin V was quantified. Phosphatidylserine is a phospholipid usually found on the inner surface of the plasma membrane, but its expression inverts externally to the outer surface of the plasma membrane during early apoptosis. Phosphatidylserine binds preferentially to annexin V, which can be used as a marker of apoptosis (41). In our PBMC cultures, annexin V staining was highest at the 6-hour timepoint and decreased progressively over 48 hours (**Figure 5A**). At 6 hours, an increase in annexin V relative to the DMSO control was detected with TLR4, TLR7/8 and TLR8 agonist stimulations (**Figure 5A**, **Supplementary Figure S4**). In comparison to DMSO, TLR4, TLR7/8, TLR8 and TLR9 agonist stimulations were all significantly increased for the annexin V signal at 24 hours, with those elevations being maintained at 48 hours. Individual TLR agonists sharing the same pathway showed similar trends to one another (**Supplementary Figure S4**). When annexin V changes were compared to small and large round cell population shifts at the 24-hour timepoint, unique patterns were identified for each of the TLR agonists (**Figure 5B**). As described previously, TLR4 stimulation showed increases in small round cells and decreases in large round cells at this timepoint. However, when a correlational analysis was used to compare the levels of annexin V positive cells, we found the frequencies of small and large round cells negatively and positively correlated with annexin V expression, respectively. TLR7 and TLR9 agonists did not correlate with the changes in annexin V positivity across wells as indicated by low slope values. Interestingly, the pronounced increases in small round cells for TLR8 agonists were accompanied by a cluster of annexin V positivity in those wells that had the highest numbers of small round cells. Those same wells had the largest decrease in large round cells, suggesting that the compositional shift favoring small round cells with TLR8 pathway stimulation could be due to the death of large round cells.

Another conventional hallmark of cell death is nuclear fragmentation, an event that occurs much later in apoptosis than phosphatidylserine expression inversion to the exterior plasma membrane, and which is also associated with other forms of cell death (42, 43). Therefore, we quantified PBMCs with fragmented nuclei in response to different TLR agonist stimulations over time from our high content images. As opposed to annexin V staining, which decreased over 48 hours, the number of cells with fragmented nuclei grew in number during the time course (**Figure 5C**). Significant increases in this cell death marker relative to DMSO were found at the 48-hour timepoint for TLR4, TLR7/8 and TLR8 agonists, a result that corresponded with annexin V expression at the 6-hour timepoint. We also detected a marked decrease in fragmented nuclei at 48 hours for TLR9 agonists compared to the DMSO control. When fragmented nuclei changes were compared to the fraction of small round cells at the 48-hour timepoint, we found that all TLRs trended upward with positive slopes (range 0.02266 to 0.03142) (**Figure 5D**). We observed the opposite effect for fragmented nuclei correlations relative to the proportion of large round cells (negative slopes ranging from –0.06132 to –0.08836).

### Phagocytosis readouts are prominently featured with TLR7 and TLR8 agonists

**Figure 6:**
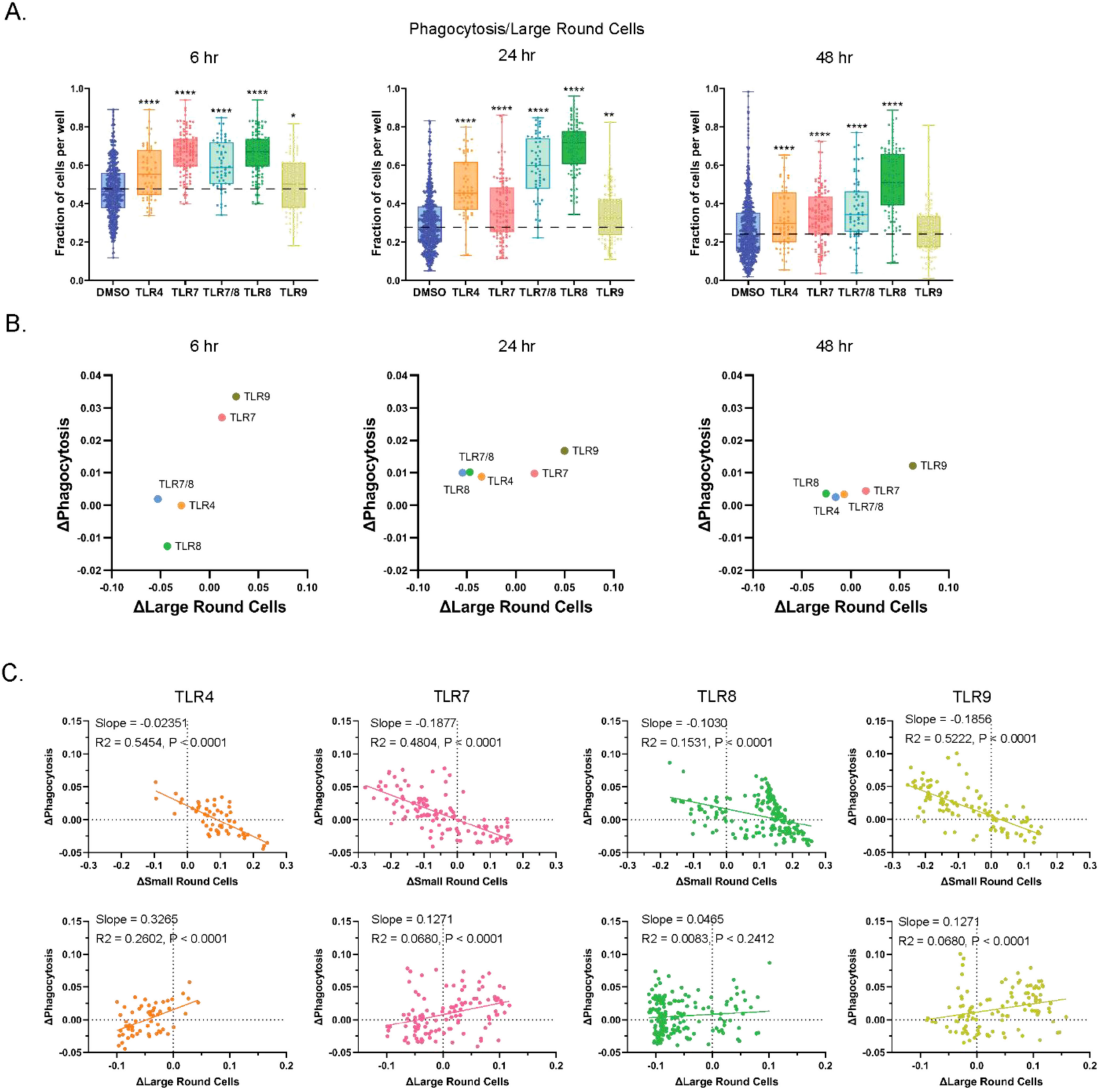
Incidence of phagocytosis is increased with TLR pathway stimulation. **(A)** Fraction of large round cells over all round large cells exhibiting phagocytosis. Data are shown for 6, 24, and 48 hrs of treatment. **(B)** Relative phagocytosis changes in TLR MOAs to DMSO in fractions of large round cells in PBMC cultures at 6, 24, and 48 hrs of culture. **(C)** Changes relative to DMSO in fragmented nuclei positivity of small and large round cells in cultures treated with TLR agonists. Statistical comparison relative to DMSO was done using a Mann-Whitney U test, **** *p* < 0.0001, ** *p* < 0.01, * *p* < 0.05, while correlational analyses were done using Spearman’s correlation, and slope is represented by R^2^ and *p*-values are indicated. The horizontal dotted lines in **(A)** represent DMSO average, with the means, minimum and maximum observation indicated by error bar, while the boxes represent the interquartile range.

Antigen-presenting cells protect the body by engulfing foreign particles, bacteria, and dead or dying cells in response to external signals, including those that activate TLRs (4), through a process called phagocytosis. Within PBMCs, larger cells (including macrophages and monocytes) perform this function. Using high-content imaging, we quantified cells with phagocytic morphology *in vitro* in healthy PBMC cultures treated with or without TLR agonists (**Figure 1B**). Phagocytic cell fractions were normalized to those of large round cells per well and reported as ‘phagocytosis/large round cells’. The proportions of phagocytosis/large round cells were highest at the 6-hour timepoint and declined over time (**Figure 6A**, **Supplementary Figure S5**). The pattern of phagocytic activity was consistent with those from previous reports (44, 45). As early as 6 hours, an increase in phagocytosis on large round cells was detected for all TLR agonists relative to DMSO, with the largest changes resulting from TLR7 and TLR8 stimulation. Elevations were maintained throughout the time course, except for TLR9 agonists at the 48-hour timepoint. Shifts in the fraction of phagocytic (y-axis) or large round (x-axis) cells relative to DMSO were plotted for each TLR agonist at each timepoint to compare MOA response patterns (**Figure 6B**). Similar to what was observed in our cell composition and death analyses, we found that TLR7 and TLR9 agonists clustered together and TLR4 and TLR8 agonists clustered together, and this effect was most pronounced at the 6-hour timepoint. When phagocytosis changes were compared to small and large round cell changes at the 24-hour timepoint for each well, a positive association was detected between increased phagocytosis and increased large round cells (**Figure 6C**). Overall, as the proportion of small round cells increased, the amount of phagocytosis decreased, and the opposite was true as populations shifted to favor large round cells. This is evident for TLR9 agonists which previously had increased the relative proportion of large round cells (**Figure 4A**). However, all TLR agonist pathways exhibited a negative slope for phagocytosis when compared to small round cells and a positive slope when compared to large round cells, this is consistent with cellular functions of the immune cell types in PBMC cultures that are known to perform phagocytosis.

### Effect of TLR stimulation on markers of cell motility

**Figure 7:**
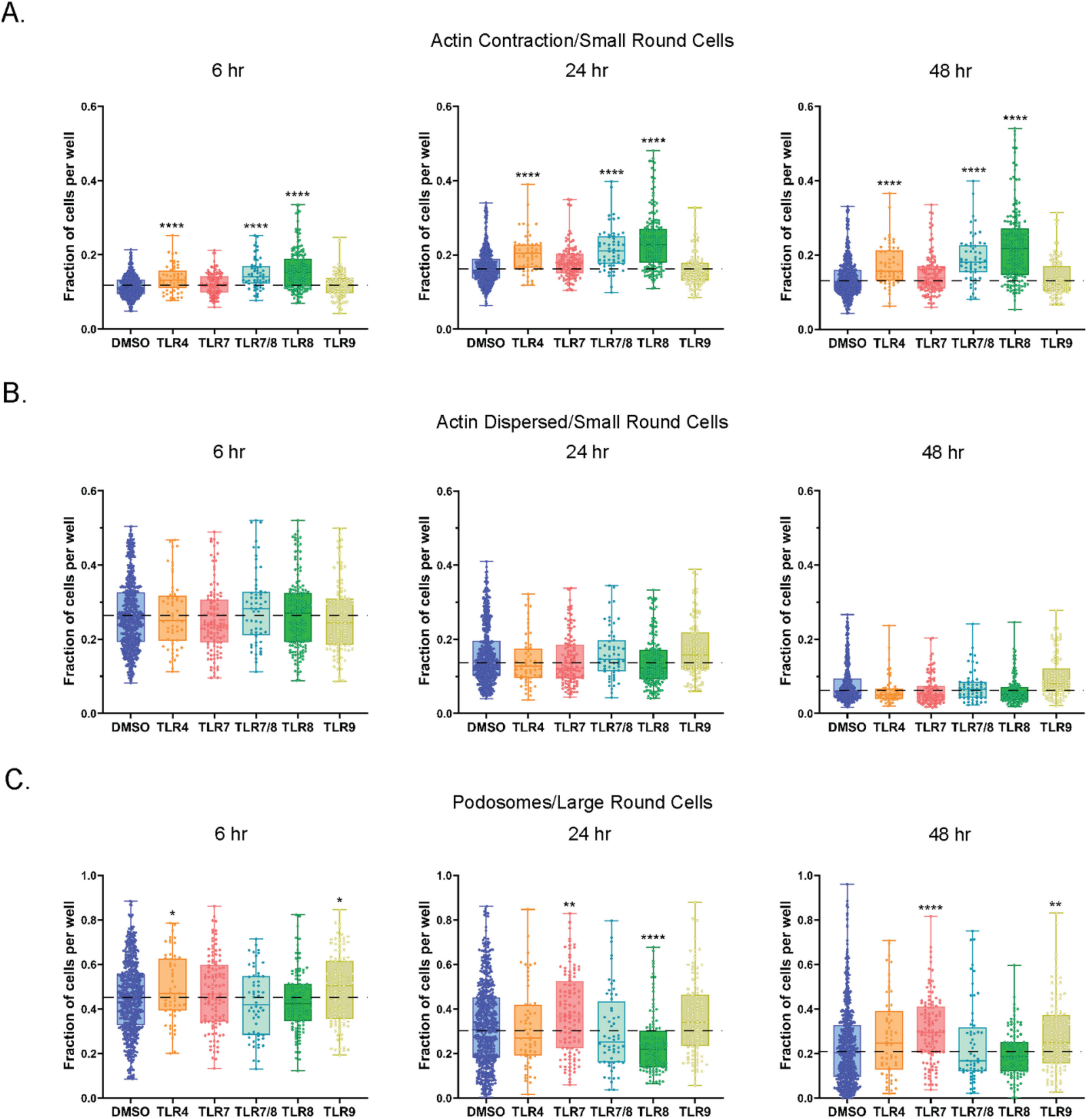
TLR agonists promote features of cell movement in small and large round cells. **(A)** Fraction of small round cells with actin contraction. Data are shown for 6, 24, and 48 hrs of treatment. **(B)** Fraction of small round cells with dispersed actin. Data are shown for 6, 24, and 48 hrs of treatment. **(C)** Fraction of podosome positive large round cells. Data are shown for 6, 24, and 48 hrs of treatment. Statistical comparison relative to DMSO was done using a Mann-Whitney U test, ****p < 0.0001, **p < 0.01, *p < 0.05. The horizontal dotted lines in **(A-C)** represent DMSO average, with the means, minimum and maximum observation indicated by error bar, while the boxes represent the interquartile range.

To determine if TLR stimulation influenced actin dynamics in stimulated cells, we first trained single cell models to recognize cells with contracted or distributed cytoskeletons as markers of movement or immobility, respectively. We then applied the trained models to quantify small round cells that were positive for motility features in healthy PBMCs (**Figure 7**). Of note, not all small round cells stained positive for actin. Detection of actin contraction indicated that TLR4 and TLR8 stimulations had a significantly higher proportion of small round cells with the contracted actin feature (**Figure 7A**). These changes were maintained at 24- and 48-hours with the greatest amplitude observed for TLR8 agonists (median difference vs DMSO of 1.55- and 1.91-fold higher, respectively). There was no observed effect of TLR7 or TLR9 agonists on actin contraction. The disassembly of actin filaments is associated with cells that are not moving. However, the pool of actin filaments is maintained if a stimulus for cell movement is received (46, 47). We found that small round cells with dispersed actin decreased over time and most TLR stimulations did not change this feature when compared to the DMSO control (**Figure 7B**). The only exception was with TLR9 agonists at the 48-hour timepoint, which did retain more actin dispersed small round cells than the DMSO control, although still at lower levels relative to TLR9 at earlier timepoints. Overall, individual TLR agonists that utilized the same signaling pathway exhibited remarkably similar effects on actin contraction and dispersion (**Supplementary Figure S6A, B**).

Podosomes are another example of actin-rich structures that are present at the periphery of the plasma membrane on a multitude of migrating cell types, including certain immune cells, such as macrophages and monocytes (48). Podosomes commonly display a polarized pattern on migrating cells, usually located on the leading edge of a migrating cell. In our study we observed higher levels of podosome positive large round cells treated with TLR4 and TLR9 agonists at the 6-hour timepoint (**Figure 7C**, **Supplementary Figure S6C**). Additionally, TLR7 agonists increased the proportion of large round cells with podosomes at both the 24- and 48-hour time points. We also detected lower levels of podosome positive large round cells following TLR8 stimulation, but only at 24-hours.

### PLWH on ART lower cytokine/chemokine PBMC responses to different TLR agonists

Next, we quantified and compared the cytokine and chemokine responses by PBMCs from healthy volunteers (HV) and PLWH on ART, following 6- and 48-hour stimulations with TLR4, TLR7, TLR7/8, TLR8 or TLR9 agonists. Cell culture supernatants from stimulated or unstimulated (DMSO) PBMCs were collected for use in quantifying 51 secreted proteins. All TLR-stimulated secreted protein responses were normalized to DMSO control.

**Figure 8:**
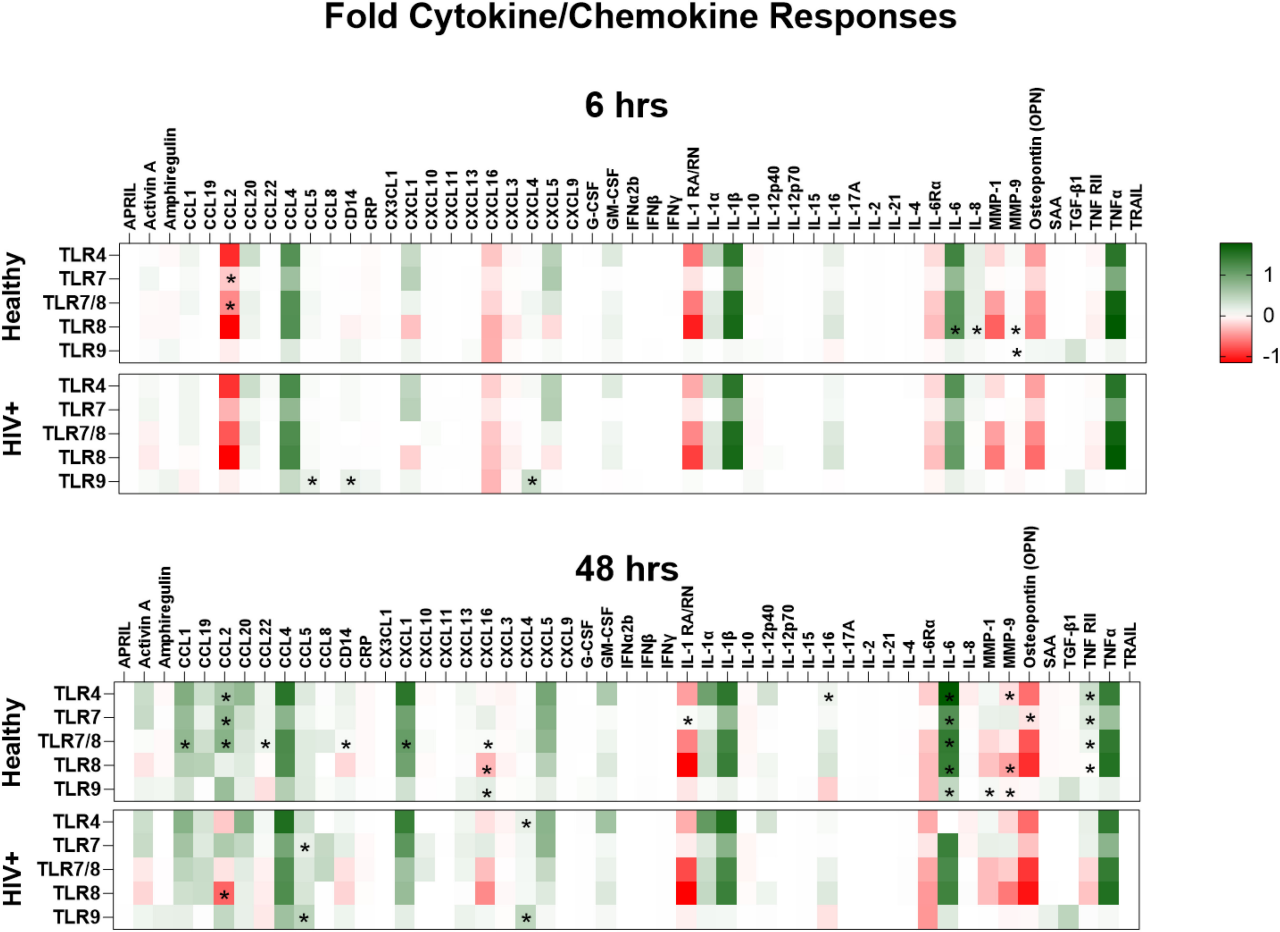
Lower inflammatory response by HIV positive PBMCs except for HIV co-receptor ligands. A comparison of the cytokines and chemokines responses by PBMCs from healthy and HIV positive donors following stimulation with TLR4 (*E. coli* LPS), TLR7 (imiquimod or vesatolimod), TLR7/8 (resiquimod), TLR8 (selgantolimod or motolimod), TLR9 (CpG 2395 or CpG 2006) are plotted into heatmaps. Cytokines and chemokines were quantified from the supernatants using a multiplex bead panel. Statistical comparison of cytokine and chemokine responses to the different TLR stimulations in PBMCs of healthy volunteers and people living with HIV on ART was done using Mann-Whitney test, and all *p*-values *p*<0.05 are indicated using asterisks * placed on the group with the higher response.

Overall, secreted protein responses in the supernatants of PBMCs of PLWH on ART were frequently lower following TLR stimulations when compared to those of HV, except for soluble CD14 (sCD14), CCL5 and CXCL4. Notably, CCL5 and CXCL4 are ligands for HIV co-receptor CCR5 (**Figure 8**, **Supplementary Figures S7**, **S8**). Similar increases in levels of HIV co-receptor ligand in blood acts as surrogate markers for HIV infection (49). PBMCs of PLWH at 6 hours, resulted in lower MMP3 responses with TLR4 stimulation and lower CCL2 levels with either TLR7 or TLR7/8 stimulations. However, higher CRP levels were found with only TLR7/8 stimulation when compared to HV (**Figure 8**, **Supplementary Figure S7**). Similarly, 6 hours of TLR8 stimulation led to lower Activin A, CCL1, CCL2, IL-1β, IL-6, IL-8, and MMP-9 responses, but higher sCD14 and CRP responses in PBMCs of PLWH in comparison to HV. CCL1 and MMP-9 responses were lower, while CCL5 and CXCL4 responses were increased in PBMCs of PLWH after 6 hours of TLR9 stimulation.

Longer TLR stimulation of PBMCs (48 hours) resulted in more striking differences in secreted protein responses between HV and PLWH (**Figure 8**). Specifically, PBMCs from PLWH at 48 hours of TLR4 stimulation had lower CCL2, IL-16, IL-6Rα, and TNF-RII responses, but higher CXCL4 production than HV. Moreover, PBMCs of PLWH produced lower amphiregulin, CCL2, IL-1RA/RN, IL-6Rα, osteopontin and TNF-RII, but higher CCL5 and CXCL4 responses to TLR7 stimulation than HV, while 48 hours of TLR7/8 stimulation resulted in lower CCL1, CCL2, CCL22, CXCL1, CXCL5, CXCL16, IL-6Rα, MMP-9, sCD14 and TNF-RII responses (**Figure 8**). A dichotomy was observed in TLR8 responses after 48 hours stimulation, with lower activin A, CCL19, CXCL16, IL-1RA/RN IL-16Rα, MMP-9 and TNFRII responses, but higher CCL2 responses in PBMCs of PLWH compared to controls. Opposing response patterns were also observed after 48 hours of TLR9 stimulation, with lower CCL1, CCL2, CXCL16, IL-6Rα, IL-6, and MMP-1 responses, but higher amphiregulin, CCL5 and CXCL4 responses by PBMCs of PLWH compared to HV (**Figure 8**, **Supplementary Figure S8**).

Overall, secreted protein responses induced by the different TLR pathways were lower in PBMCs from PLWH, except for sCD14, CCL2, CCL5 and CXCL4, which were higher in response to TLR4, TLR7 and TLR9 stimulations, and CRP, which was higher in PLWH only after early TLR7 and TLR8 stimulation. As CCR5 is the cognate ligands from HIV co-receptor, binding CCL5 these increased responses could be relevant to overall pathogenesis.

### TLR agonist dependent differences in cellular frequencies and cytological features are observed for HV and PLWH PBMC cultures

Using the same high content imaging, features defining cellular size, cell motility, and nuclear shape, were analyzed from PBMCs of HV and PLWH with TLR4, TLR7, TLR7/8, TLR8, or TLR9 stimulations after 6, 24, and 48 hours of stimulation. The resulting high-content images were analyzed using the same machine-learning trained algorithms described above (**Figure 1B**). The results were plotted as color-coded heatmaps representing the fraction of cells per well that were positive for each listed feature or normalized using previously described criteria (**Table 2**), with significant changes relative to DMSO indicated (**Supplementary Figure S9**).

**Table 2:** Normalization scheme for cell features.

Cell Feature	Normalization
Small round cells	None
Large round cells	None
Annexin V	None
Phagocytosis	$\text{Normalized}\;=\;\frac{\text{Phagocytosis}}{\text{Phagocytosis}\;+\;\text{Large}\;\text{round}\;\text{cells}}$
Fragmented nucleus	None
Actin contracted	$\text{Normalized}\;=\;\frac{\text{Actin}\;\text{contracted}\;\text{lymphocytes}}{\text{Actin}\;\text{contracted}\;\text{lymphocytes}+\text{Actin}\;\text{dispersed}\;\text{lymphocytes}}$
Actin dispersed	$\text{Normalized}\;=\;\frac{\text{Actin}\;\text{dispersed}\;\text{lymphocytes}}{\text{Actin}\;\text{contracted}\;\text{lymphocytes}+\text{Actin}\;\text{dispersed}\;\text{lymphocytes}}$
Large cell podosomes	$\text{Normalized}\;=\;\frac{\text{Large}\;\text{cell}\;\text{podosomes}}{\text{Large}\;\text{cell}\;\text{podosomes}+\text{Large}\;\text{cell}\;\text{no}\;\text{podosomes}}$
Large cell no podosomes	$\text{Normalized}\;=\;\frac{\text{Large}\;\text{cell}\;\text{no}\;\text{podosomes}}{\text{Large}\;\text{cell}\;\text{podosomes}+\text{Large}\;\text{cell}\;\text{no}\;\text{podosomes}}$

**Figure 9:**
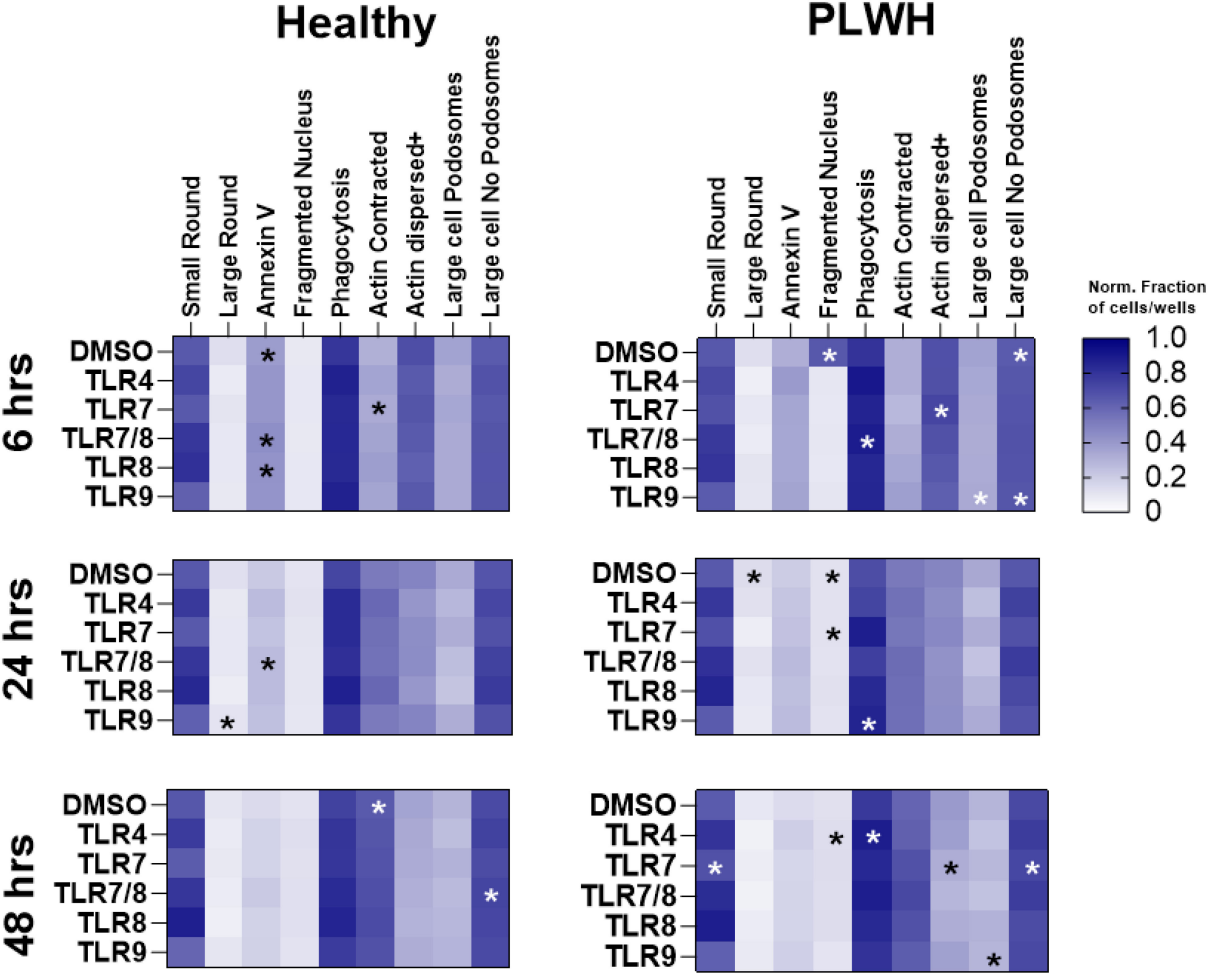
Differences in cytological features between PBMCs from health donors and people living with HIV. Heatmaps of cell features obtained confocal imaging and analyzed using machine learning. Various cell features were determined and used to characterize cells into small or large immune cells, annexin V positive, cells with fragmented nucleus, cell with contracted or dispersed actin, or with or without podosomes, PBMCs from healthy donors (n=20) and people living with HIV (n=5) were stimulated with TLR4 (*E. Coli* LPS), TLR7 (imiquimod or vesatolimod), TLR7/8 (resiquimod), TLR8 (selgantolimod or motolimod), TLR9 (CpG 2395 or CpG 2006). Quantification of the different cell features is based on fraction of cells normalized using scheme in **Table 2**. Statistical comparison was done using Mann-Whitney test, and all *p*-values *p*<0.05 are indicated using asterisks * placed on the group with the higher response.

Changes in the cell features were heterogeneous in the two groups (**Figure 9**, **Supplementary Figure S9**). Six hours of TLR7 stimulation resulted in a lower fraction of actin dispersed cells, but a higher fraction of actin contracted cells in PBMCs of PLWH compared to HV. A smaller fraction of cells was positive for annexin V, but a more substantial fraction of large round cells was undergoing phagocytosis in PBMCs of PLWH following TLR7/8 stimulation than HV. Following overnight stimulation with TLR7, a greater increase in the fraction of cells with fragmented nuclei in PBMCs of PLWH was observed compared to those of healthy controls. In contrast, 48 hours of TLR7/8 stimulation resulted in a reduced fraction of annexin V positive cells in PBMCs of PLWH than HV. After 24 hours, there was a significantly lower number of large round cells but a higher number of large round cells undergoing phagocytosis in PBMCs of PLWH with TLR9 stimulation. Moreover, 48 hours of TLR4 stimulation led to larger fractions of fragmented cells and phagocytosis, while TLR7 stimulation resulted in higher frequencies of small round cells, higher fractions of PBMCs with dispersed actin and large round cells with no podosomes, in PBMCs of PLWH compared to healthy controls. 48 hours of TLR7 stimulation also led to higher numbers of large round cells lacking podosomes, while 6 and 48 hours of TLR9 stimulation resulted in a higher fraction of large round cells with and without podosomes in PBMCs from PLWH compared to controls (**Figure 9**). Interestingly, unstimulated PBMCs from PLWH also had higher numbers of cells with fragmented nuclei and large round cells lacking podosomes, but fewer cells were positive for annexin V and actin contraction between 6-24 hours of culture (**Figure 9**, **Supplementary Figure S9**).

Overall, the image analysis of cell features revealed a heterogeneity in responses in the two study groups, with significant differences being observed in the proportions of cells undergoing phagocytosis, nuclear fragmentation and actin contraction or dispersal.

## Discussion

In complex cell culture experiments involving mixed populations of immune cells, an enormous amount of data can be extracted using high content image analysis, with the dimensionality of the data being amplified through the inclusion of plasma membrane, cytoplasmic, and nuclear features. The rich information provided by such images is particularly well-suited to machine-learning based analytical approaches, which can parse, compare, and reduce large, complex datasets based on known and unknown image features. This study used an ML/AI based analysis of high-content images to identify and quantify cytological features in mixed cell populations of PBMCs stimulated with different TLR agonists, in combination with matched cytokine/chemokine supernatant profiling. It allowed for the discovery of biologically interpretable features that are uniquely promoted by distinct TLR pathways, a strategy which can be used to expand the understanding of innate responses in healthy and diseased states. Specifically, we utilized deep learning-based algorithms (StarDist) to detect single cells and then applied convolutional neural networks to extract image embeddings from single cell image crops. The embeddings arising from custom-built supervised learning tools (utilizing XGBoost), were used to quantify different cell features, and to resolve differences within and across many high-content images (23).

This combined approach was able to distinguish between the responses generated by small round cells (lymphoid-like), and large round cells (myeloid-like) to different types of TLR stimuli. Specifically, it uncovered an increase in the proportions or small/lymphoid-like cells in response to TLR4 and TLR8 agonists, and a decrease in the frequencies of the same in response to TLR7 and TLR9 agonists over the course of healthy PBMC cultures. On the contrary, decreases in proportions of large round/myeloid-like cells were observed with TLR4, TLR7/8 and TLR8, whereas the proportions of the same population remained unchanged with TLR7 but increased with TLR9 stimulations, where responsiveness to TLR agonists is dependent on cellular expression of the cognate receptors. Of the cells found in PBMCs, TLR4 has been shown to be highly expressed on monocytes/macrophages, DCs, and NK cells and lowly on CD4+ T cells and B cells (50, 51). TLR7 is expressed primarily on pDCs and B cells, but also on monocytes/macrophages (52, 53) while TLR8 is expressed on monocytes and mDCs (54). Based on the size and changes in proportions of small/lymphoid-like to TLR4 and TLR8, we inferred that these cells potentially included T and B cells. While TLR7 and TLR9 responding large/myeloid-like cells, based on the changes in proportions and size, were inferred to be largely monocytes differentiated to adherent macrophages, but also included the sparse pDCs.

Several studies have evaluated TLR expression and functions in PBMC cultures in both healthy and PLWH donor PBMCs, but to our knowledge this is the first study to directly compare the cytological features induced by distinct TLR pathways using AI analysis of high-content images and cytokine expression profiles from the same set of donors. TLR4, TLR7/8, and TLR8 stimulations led to a higher proportion of small round/lymphoid-like cells expressing annexin V and having more fragmented nuclei, indicating these cells had increased motility (fragmented nuclei) while undergoing apoptosis. Moreover, the proportion of small round/lymphoid like cells correlated with fragmented nuclei, suggesting the smaller cells that also had higher actin contraction had greater motility in culture consistent with previously described highly motility in T cells (55). In contrast the large round/myeloid-like cells which the findings of this study suggest are monocytes and B cells, have previously been found to have slower motility in culture, and the slower motility being ascribed to increased adhesion to extracellular matrices (56). This was supported by the higher phagocytosis observed in large round/myeloid-like cells early in culture (6 hrs) that reduced over time, with the degree of phagocytosis of TLR7 and TLR9 stimulated cells clustering together, while those of TLR4, TLR7/8 and TLR9 clustering separately. In addition, the proportion of large round cells/myeloid-like cells positively correlated with the degree of phagocytosis, whereas the proportion small round/lymphoid-like cells negatively correlated with the same. Within the first few hours of stimulation, TLR4 and TLR8 cellular features were clustered together but separated from each other by 48 hours of culture. A similar trend was observed for TLR7 and TLR9 agonists despite the known differences in expression of these TLRs on different cells across PBMC cell types, except B cells and plasma B cells. It is important to note that TLR7 and TLR9 were highly expressed in pDCs but these cells are not highly represented in PBMC cultures.

An immunological assessment of soluble protein- cytokines, chemokines, growth factors and secreted proteins, revealed that TLR stimulation resulted in the previously observed cytokine and chemokine responses profiles that were highly specific to cognate TLR agonists. For example, TLR4, TLR7, and TLR8 stimulation of HV PBMCs increased CCL4, IL-1β, IL-6 and TNFα levels at 6 and 48 hours consistent with previously described pro-inflammatory responses by monocytes among other cell types (57–60). The only TLR which we found is not expressed in peripheral monocytes is TLR9 and consequently pro-inflammatory factors were not increased with TLR9 agonists in our study. There were several differentially regulated cytokine examples which could be explained by TLR expression in individual cell types. CCL2 was increased by TLR4 and TLR8 agonists at 6 hours but had returned to baseline by the 48-hour timepoint for all TLR agonists. Taken together, all the TLR agonists evaluated enhanced expression of both cytokines and chemokines, with the cellular size and responsiveness defined by the changes in expression of cellular features extracted using a ML/AL from high-dimensional images. The connection of these two data sets was useful for inferring responding cell-types.

This combined approach was used to compare the responses of PBMCs from PLWH and HV. Overall, the different TLR agonist stimulations resulted in lower cytokine and chemokine responses by PBMCs from PLWH when compared to HV, except for sCD14, CCL5 and CXCL4, which are surrogate markers from HIV infection or cognate chemokines for HIV’s co-receptor CCR5. The expression of these factors was higher in cell culture supernatants from PLWH stimulated with TLR4, TLR7 or TLR9. The differences in responsiveness between PBMCs of HV and PLWH was most evident at 48 hours of stimulation. We found that PBMCs from healthy and HIV positive donors had similar single cell TLR expression patterns, suggesting that TLR expression itself was likely not responsible for the differences observed. Furthermore, the image analysis using the ML/AI tool revealed that HIV infection dysregulates the patterns of expression of cellular features by increasing nuclear fragmentation, phagocytosis, and motility-dispersal actin and podosome changes, a manner that differed from HV. Together, these observations highlighted the HIV driven dysregulation of cytological changes in response to different TLR stimulations which adds to the knowledge in the field. The lower cytokine and chemokine responses by the PBMCs from ART treated individuals observed in this study, are consistent with reduction in TLR responsiveness, expression, and downstream signaling that has previously been described in ART-treated individuals (19, 61, 62).

As therapeutic strategies continue to explore the use of TLR agonists to treat disease, it is important to understand their potential benefits and limitations. This includes the downstream characterization of these agonists on unique cell populations and phenotypes. Here, we used a hypothesis-driven approach to select previously identified phenotypes and features to train single cell ML models and automate quantification across a large imaging dataset. This experimental system captures general cell biological features and morphological profiles that could be used for hypothesis generation or hypothesis-agnostic analyses. Specifically, unsupervised ML/AI approaches can be applied to discover entirely new features that are associated with TLR agonism which can help to determine how TLR signaling may be modulated to resolve both infectious and autoimmune diseases. The challenge will be in interpreting the biological importance and relevance of those new features, which may not always be obvious. We also recognize that there may be other limitations to this approach, including the elucidation of *in vitro* data as it may apply *in vivo*. Nevertheless, experiments such as these will provide new and useful information to further understand treatment and disease.

## Data Availability

The datasets presented in this study can be found in online repositories. The names of the repository/repositories and accession number(s) can be found below: https://doi.org/10.5281/zenodo.14226534.

## Supplementary material

Supplementary Figure 1 Quality of image-based experiments. **(A)** Cell counts per sample. **(B)** Statistical confidence in our identified phenotypes and assay quality scoring. DMSO wells were classified as the negative control, and motolimod at 5µM was taken as the positive control.

Supplementary Figure 2 TLR expression from bulk RNAseq of immune cells sorted from PBMCs of healthy donors. Transcript expression values calculated as nTPM (normalized transcripts per million), resulting from whole blood of 6 *healthy* donors sorted by flow cytometry into 18 immune cell types (2).

Supplementary Figure 3 TLR4 and TLR8 agonists promote the accumulation of small round cells. **(A)** Quantification of small round cells at 6, 24, and 48 hrs of culture with DMSO or TLR agonists. Data is shown as a fraction of total small round cells per well. **(B)** Measurements of large round cells at selected timepoints after DMSO or TLR agonist treatment. Data is shown as fraction of total large round cells per well. Statistical comparison relative to DMSO was done using a Mann-Whitney U test, *****p* < 0.0001.

Supplementary Figure 4 Increases in features of cell were observed in cultures stimulated with TLR4 and TLR8 agonists. **(A)** Fraction of annexin V+ cells per well in DMSO control and TLR agonist stimulated cell cultures. Data are shown for 6, 24, and 48 hrs of treatment. **(B)** Changes relative to DMSO in annexin V positivity of small and large round cells in cultures treated with TLR agonists. Statistical comparison relative to DMSO was done using a Mann-Whitney U test, *****p* < 0.0001, ****p* < 0.001, ***p* < 0.01, **p* < 0.05.

Supplementary Figure 5 Incidence of phagocytosis is increased with TLR pathway stimulation. Fraction of large round cells exhibiting phagocytosis. Data are shown for 6, 24, and 48 hrs of treatment. Statistical comparison relative to DMSO was done using a Mann-Whitney U test,****P < 0.0001, ***P < 0.001, **P < 0.01, *P < 0.05.

Supplementary Figure 6 TLR agonists promote features of cell movement in small and large round cells. **(A)** Fraction of small round cells with actin contraction. Data are shown for 6, 24, and 48 hrs of treatment. **(B)** Fraction of small round cells with dispersed actin. Data are shown for 6, 24, and 48 hrs of treatment. **(C)** Fraction of podosome positive large round cells. Data are shown for 6, 24, and 48 hrs of treatment. Statistical comparison relative to DMSO was done using a Mann-Whitney U test, ****P < 0.0001, ***P < 0.001, **P < 0.01, *P < 0.05.

Supplementary Figure 7 Comparison of Cytokine and Chemokine responses after 6 hours of TLR stimulations. Peripheral blood mononuclear cells from healthy (squares, n=30) and people living with HIV (PLWH, triangle, n=10), were stimulated for 6 hours with TLR4 (blue bars- *E. Coli* LPS), TLR7 (orange bars-combined Imiquimod and vesatolimod), TLR7/8 (red bars-resiquimod), TLR8 (teal bars-combined selgantolimod, motolimod), TLR9 (green bars-combined CpG 2395 and CpG 2006). Cytokine and chemokines were quantified from TLR stimulated PBMC supernatants and plotted into floating bars with scatter plots. Statistical comparison between the groups was done using a Mann-Whitney U test, all *p*<0.05 indicated with asterisks * *p*<0.05-0.01 with **, *p*<0.01-0.001 with ***, and *p*<0.001.

Supplementary Figure 8 Comparison of Cytokine and Chemokine Responses after 48 hours of TLR stimulation. Peripheral blood mononuclear cells (PBMCs) from healthy (squares, n=30) and people living with HIV (PLWH, triangle, n=10), were stimulated for 48 hours with TLR4 (blue bars- *E. Coli* LPS), TLR7 (orange bars-combined Imiquimod and vesatolimod), TLR7/8 (red bars-resiquimod), TLR8 (teal bars-combined selgantolimod, motolimod), TLR9 (green bars-combined CpG 2395 and CpG 2006). Multiplex bead arrays were used to quantify cytokine and chemokines from TLR stimulated PBMC supernatants and plotted into floating bars with scatter plots. Statistical comparison between the groups was done with Mann-Whitney test, all *p*<0.05 indicated with asterisks * *p*<0.05-0.01 with **, *p*<0.01-0.001 with ***, and *p*<0.001.

Supplementary Figure 9 Peripheral blood mononuclear cells were stimulated with TLR4 (*E. coli* LPS), TLR7 (combined Imiquimod and vesatolimod), TLR7/8 (resiquimod), TLR8 (combined selgantolimod, motolimod), TLR9 (combined CpG 2395 and CpG 2006). Machine learning algorithm was used to acquire cell features from confocal images. The features were cell size- small round cells **(A)** and large round cells **(B)**-, cell death- annexin V, **(C)**, nuclear fragmentation **(D)**, phagocytosis **(E)** cytoplasmic actin contraction **(F)**, dispersal **(G)** and the presence **(H)** or absence of podosomes **(I)** PBMCs. Floating bars and scatter graphs are used to compare the cell feature data from healthy (n=30) and HIV-positive PBMCs, (n=10) with statistical comparison being done using Mann-Whitney test, and *p<*0.05 indicated using *, *p*<0.01 with **, *p*<0.001 with *** and *p<*0.0001.

Supplementary Table 1 Genes from Blood Transcription Modules (BTM) (1).
