# Supplementary material for: AI discovery of TLR agonist-driven phenotypes reveals unique features of peripheral cells from healthy donors and ART-suppressed people living with HIV
Source: Front Immunol. 2025 Mar 25;16:1541152. doi: 10.3389/fimmu.2025.1541152 (PMC11975909; doi:10.3389/fimmu.2025.1541152)
Supplement: Supplementary Figure 1 — Quality of image-based experiments. (A) Cell counts per sample. (B) Statistical confidence in our identified phenotypes and assay quality scoring. DMSO wells were classified as the negative control, and motolimod at 5µM was taken as the positive control. [file DataSheet1.docx]

# Supplementary Materials

Supplementary Table 1. Genes from Blood Transcription Modules (BTM) (1)

| **Cell type** | **Module genes** |
| --- | --- |
| CD8+ T-cells | CD8A, CD8B, CD96, CRTAM, DSC1, LY9, PCNT, TSPAN32 |
| CD4+ T-cells | BAD, CCR4, CD2, CD28, CD3G, CD40LG, CD5, CTLA4, FOXP3, GOLGA4, HIPK1, HMOX2, ICOS, LEPROTL1, NUDCD3, PLCL1, PPP2CA, SIRPG, SON, SUPV3L1, TRAT1 |
| B-cells | BLK, CD19, CD22, CD37, CD79A, FCRL2, GGA2, MBD4, MS4A1, PNOC, SMC6, SNX2, SP140, STAG3, STAP1 |
| Plasma cells | ADM2, AMPD1, CCL25, CCNC, CD79A, CNKSR1, FKBP2, HSP90B1, KCNN3, MTDH, NPAS1, PNOC, PREB, RGS13, SEC24A, SERP1, SLC5A2, SPATS2, SSR4, TMEM39A, TNFRSF17, TP73, TSHR, UBA5, VPREB3, ZBP1 |
| NK cells | AGK, CD244, FASLG, IL18RAP, KLRD1, NCR1, NMUR1, PRF1, PTGDR, PTPN4, SACM1L, TBX21, TKTL1, XCL1, ZMYND11 |
| Monocytes | ASGR2, CFP, FBXL5, FCAR, FCN1, LILRA5, MEFV, MS4A6A, S100A12, TREM1, VENTX |
| cDC | CCL17, CCL24, CD1A, CD1B, CD1C, CD1E, CD209, CD80, CLEC10A, FCER1A, GFRA2, KCNK13 |
| pDC | CD2AP, CLEC4C, CUX2, FUT7, GZMB, IDH3A, IL3RA, KCNK10, LILRA4, LILRB4, LRRC36, NRP1, P2RY14, PTCRA, SCT, SLC12A3, SPIB, TLR7, TSPAN13 |

Supplementary Figure 1. Quality of image-based experiments. (A) Cell counts per sample. (B) Statistical confidence in our identified phenotypes and assay quality scoring. DMSO wells were classified as the negative control, and motolimod at 5µM was taken as the positive control.

A.


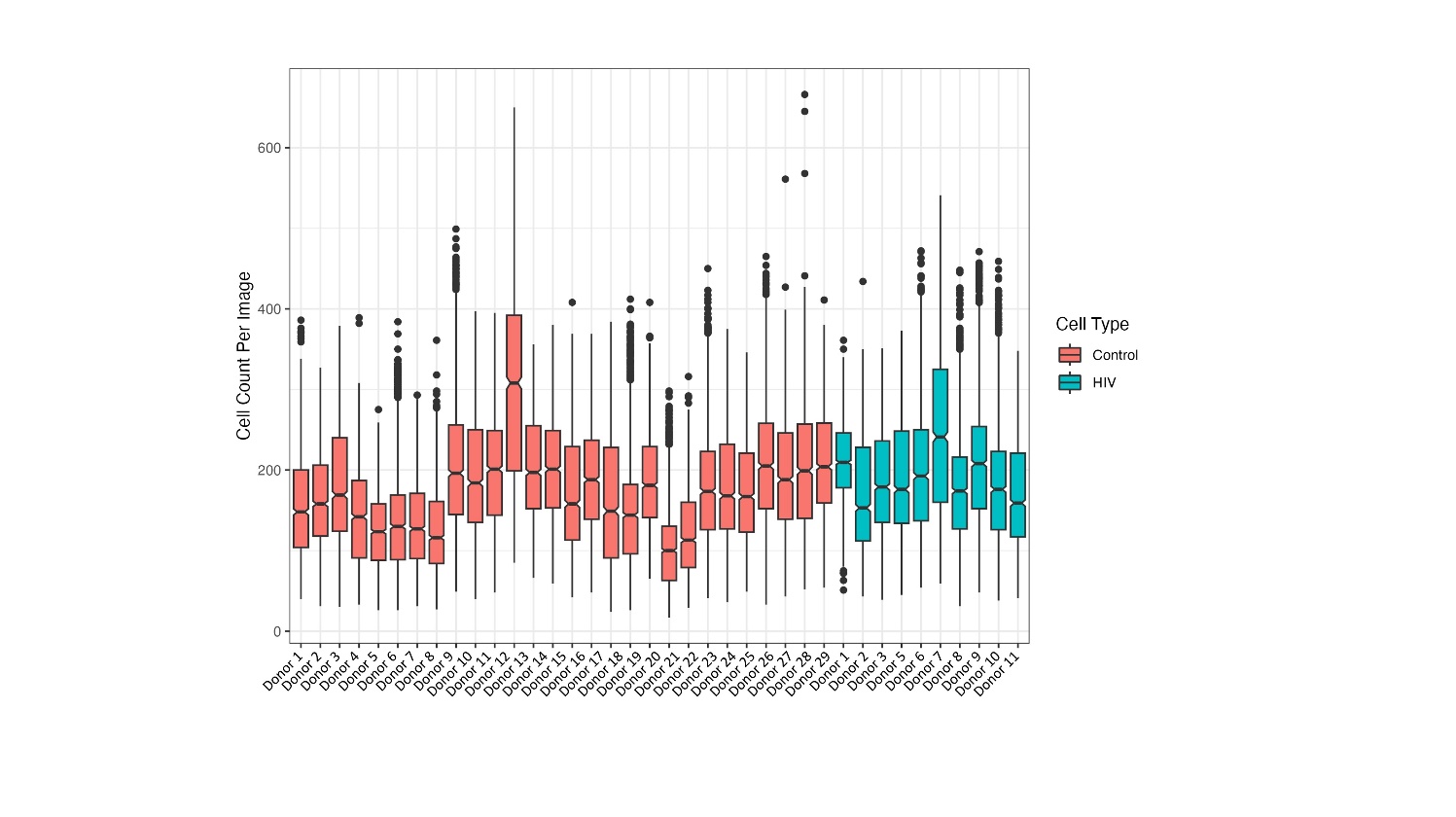


**B.**


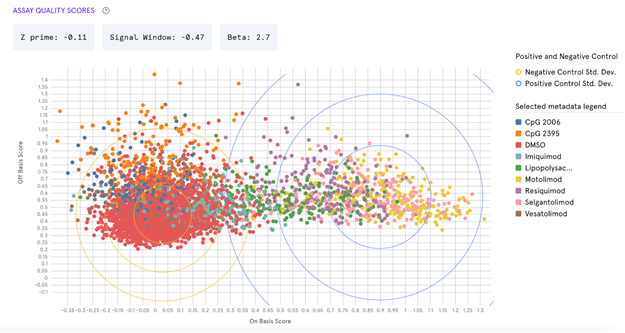


Supplementary Figure 2. TLR expression from bulk RNAseq of immune cells sorted from PBMCs of healthy donors. Transcript expression values calculated as nTPM (normalized transcripts per million), resulting from whole blood of 6 *healthy* donors sorted by flow cytometry into 18 immune cell types (2).


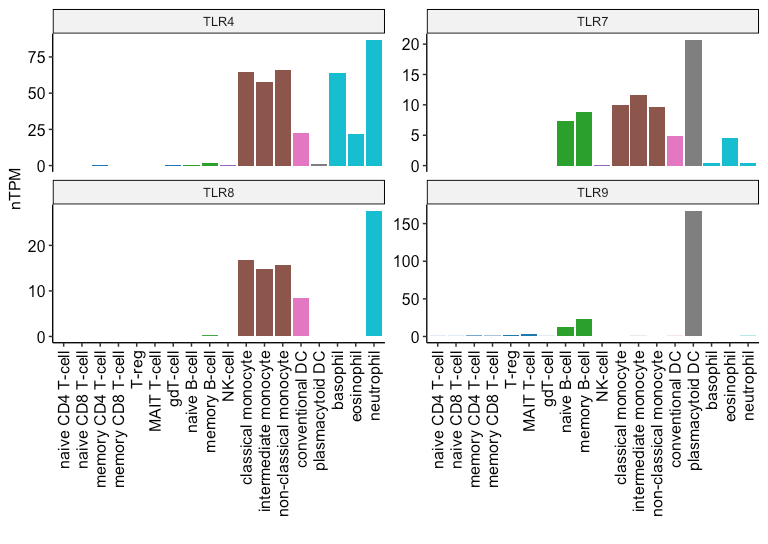


Supplementary Figure 3. TLR4 and TLR8 agonists promote the accumulation of small round cells. (A) Quantification of small round cells at 6, 24, and 48 hrs of culture with DMSO or TLR agonists. Data is shown as a fraction of total small round cells per well. (B) Measurements of large round cells at selected timepoints after DMSO or TLR agonist treatment. Data is shown as fraction of total large round cells per well. Statistical comparison relative to DMSO was done using a Mann-Whitney U test, *****p* < 0.0001.


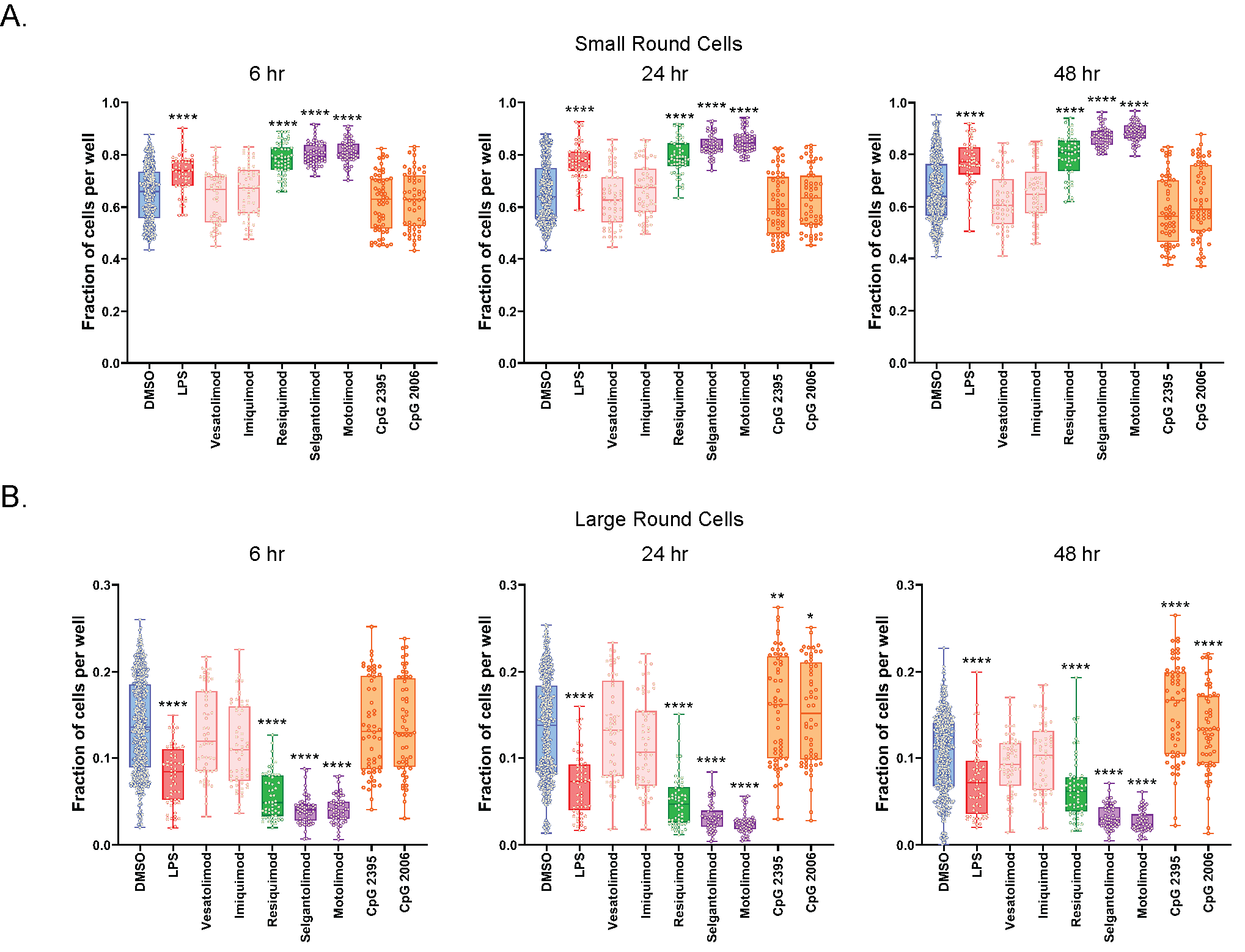


Supplementary Figure 4. Increases in features of cell were observed in cultures stimulated with TLR4 and TLR8 agonists. (A) Fraction of annexin V+ cells per well in DMSO control and TLR agonist stimulated cell cultures. Data are shown for 6, 24, and 48 hrs of treatment. (B) Changes relative to DMSO in annexin V positivity of small and large round cells in cultures treated with TLR agonists. Statistical comparison relative to DMSO was done using a Mann-Whitney U test, *****p* < 0.0001, ****p* < 0.001, ***p* < 0.01, **p* < 0.05.


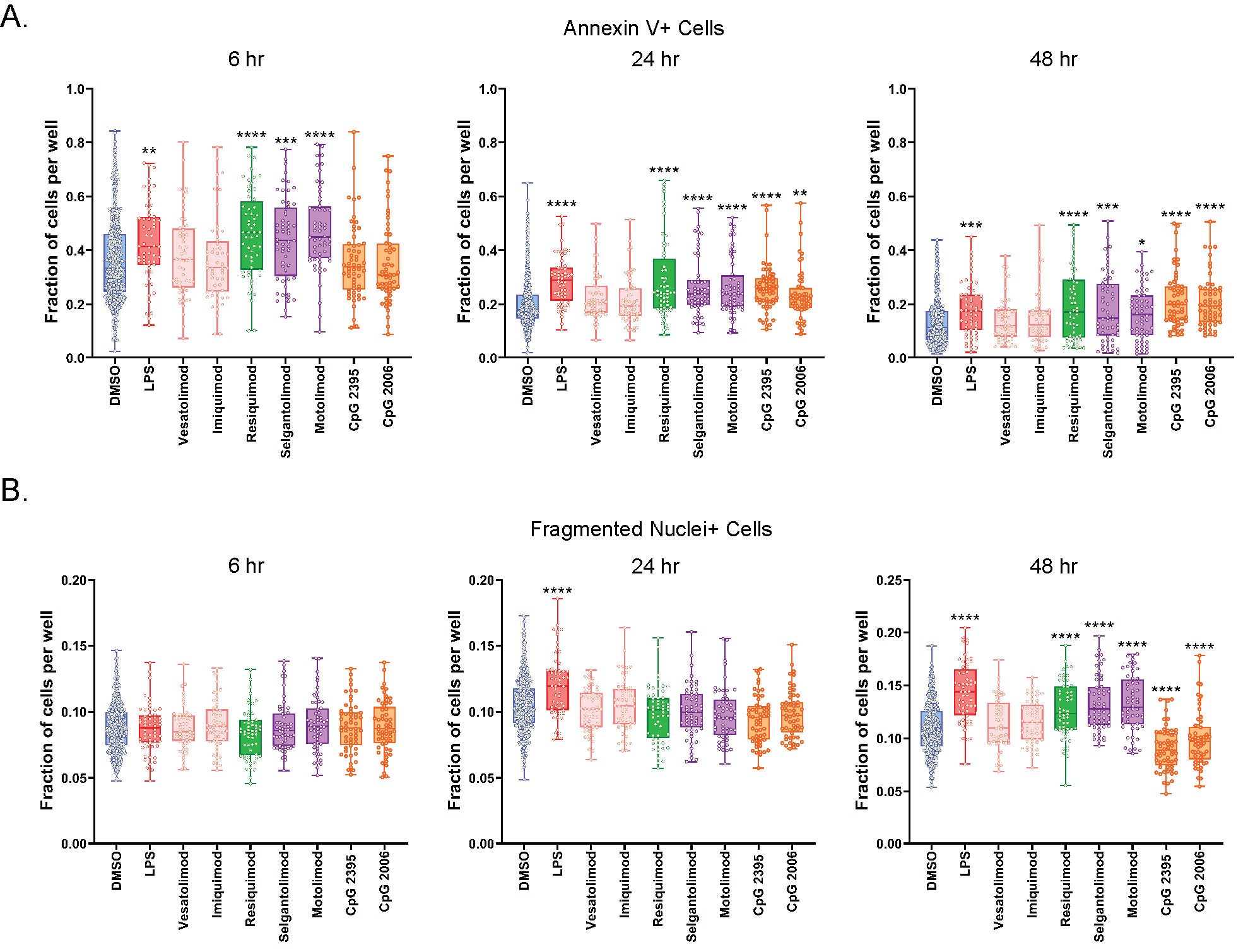


Supplementary Figure 5. Incidence of phagocytosis is increased with TLR pathway stimulation. Fraction of large round cells exhibiting phagocytosis. Data are shown for 6, 24, and 48 hrs of treatment. Statistical comparison relative to DMSO was done using a Mann-Whitney U test,****P < 0.0001, ***P < 0.001, **P < 0.01, *P < 0.05.


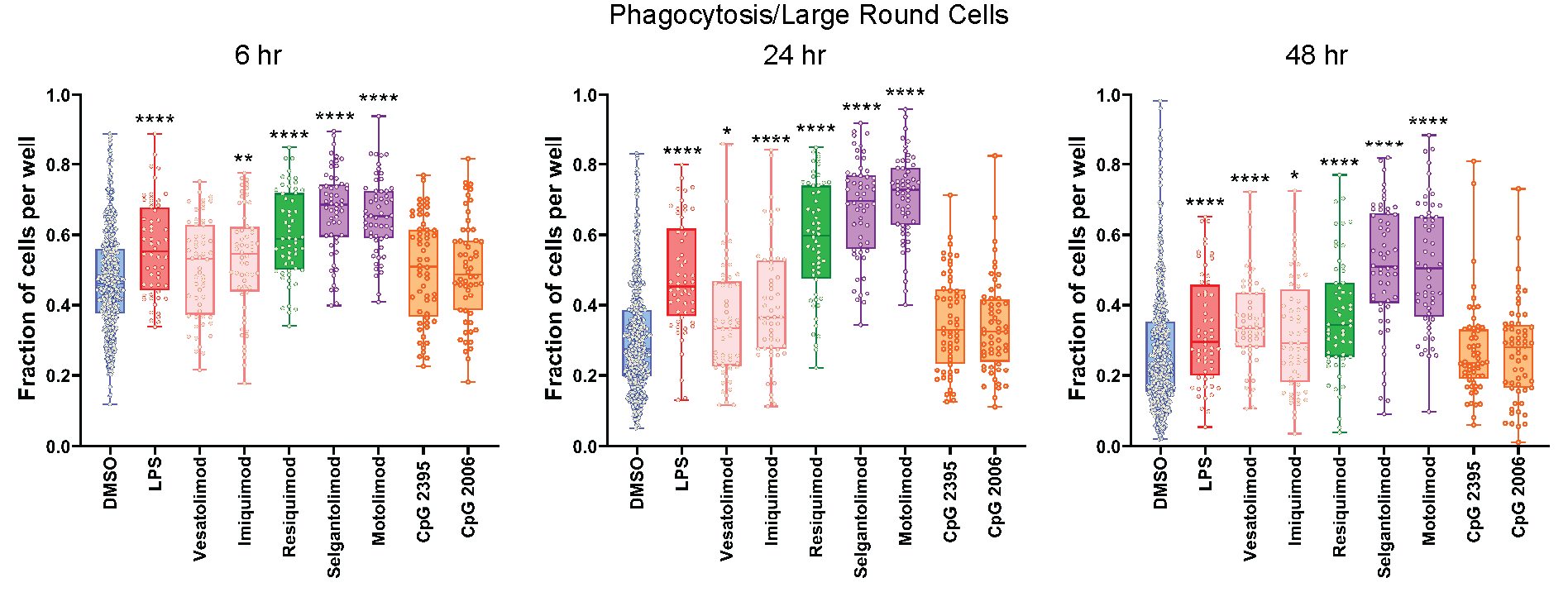


**Supplementary Figure 6. TLR agonists promote features of cell movement in small and large round cells.** (A) Fraction of small round cells with actin contraction. Data are shown for 6, 24, and 48 hrs of treatment. (B) Fraction of small round cells with dispersed actin. Data are shown for 6, 24, and 48 hrs of treatment. (C) Fraction of podosome positive large round cells. Data are shown for 6**,** 24, and 48 hrs of treatment. Statistical comparison relative to DMSO was done using a Mann-Whitney U test, ****P < 0.0001, ***P < 0.001, **P < 0.01, *P < 0.05.


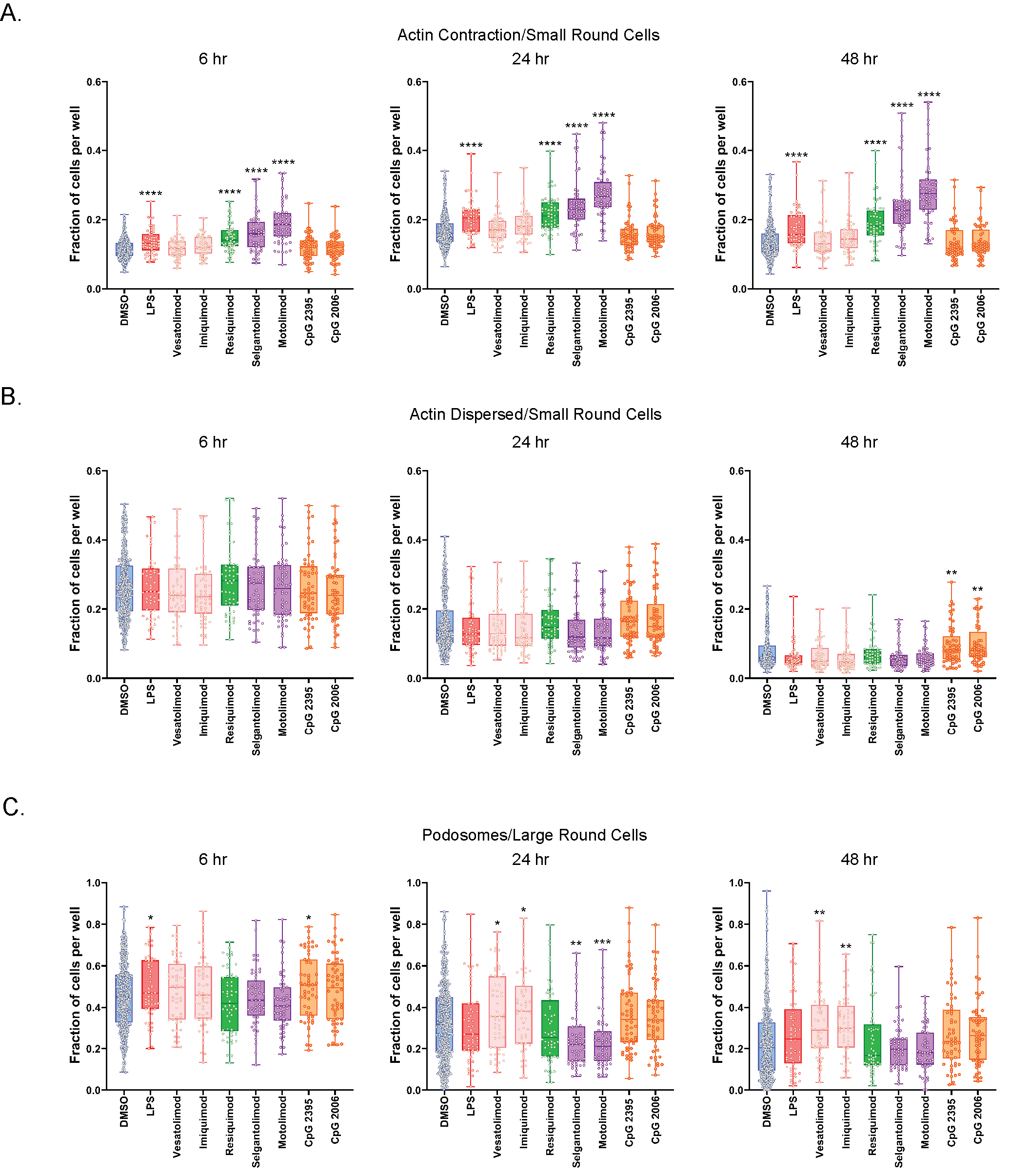


Supplementary Figure 7. Comparison of Cytokine and Chemokine responses after 6 hours of TLR stimulations. Peripheral blood mononuclear cells from healthy (squares, n=30) and people living with HIV (PLWH, triangle, n=10), were stimulated for 6 hours with TLR4 (blue bars- *E. Coli* LPS), TLR7 (orange bars-combined Imiquimod and vesatolimod), TLR7/8 (red bars-resiquimod), TLR8 (teal bars-combined selgantolimod, motolimod), TLR9 (green bars-combined CpG 2395 and CpG 2006).Cytokine and chemokines were quantified from TLR stimulated PBMC supernatants using multiplex arrays and plotted into floating bars with scatter plots. Statistical comparison between the groups was done using a Mann-Whitney U test, all *p*<0.05 indicated with asterisks * *p*<0.05-0.01 with **, *p*<0.01-0.001 with ***, and *p*<0.001.


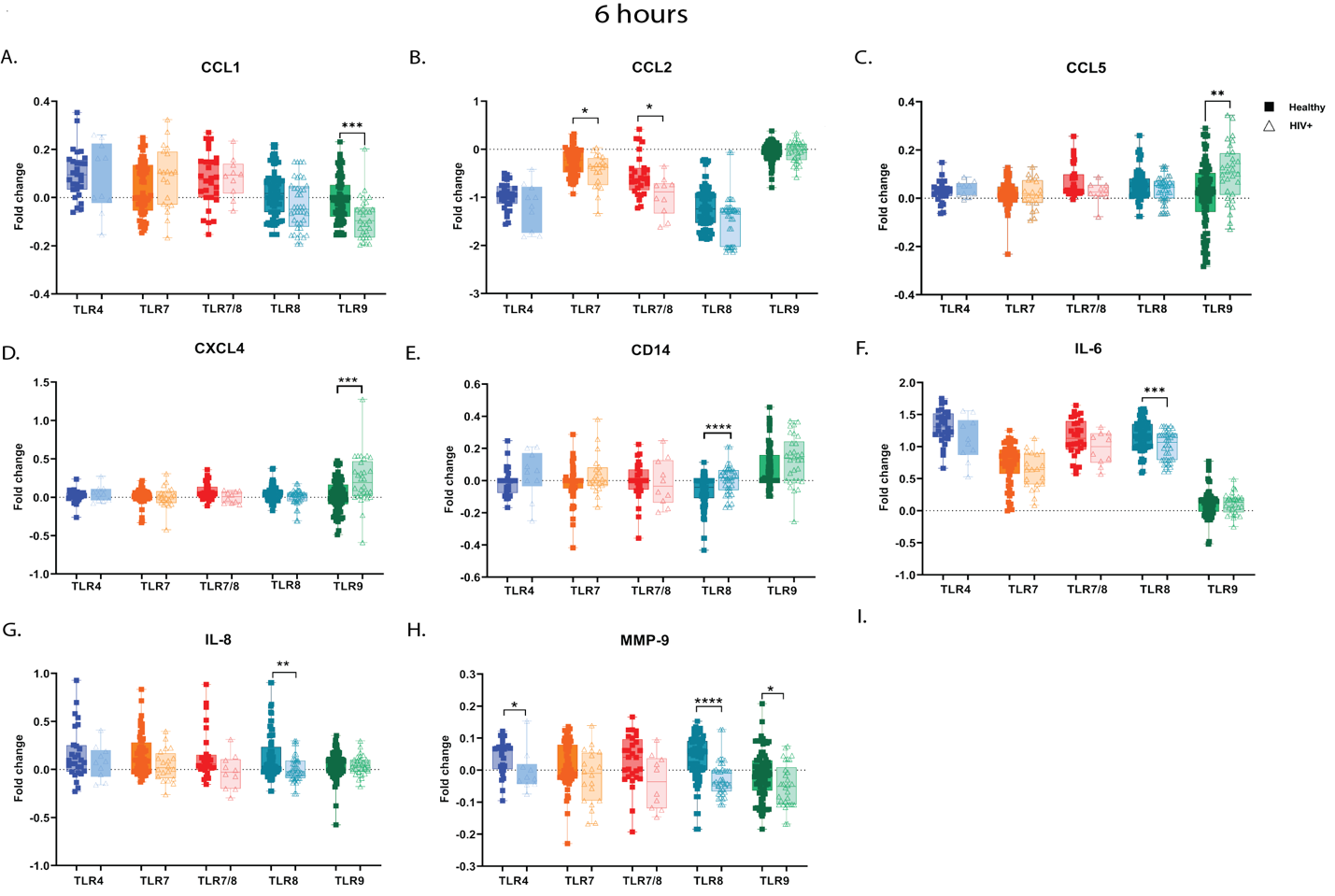


Supplementary Figure 8. Comparison of Cytokine and Chemokine Responses after 48 hours of TLR stimulation. Peripheral blood mononuclear cells (PBMCs) from healthy (squares, n=30) and people living with HIV (PLWH, triangle, n=10), were stimulated for 48 hours with TLR4 (blue bars- *E. Coli* LPS), TLR7 (orange bars-combined Imiquimod and vesatolimod), TLR7/8 (red bars-resiquimod), TLR8 (teal bars-combined selgantolimod, motolimod), TLR9 (green bars-combined CpG 2395 and CpG 2006). Multiplex bead arrays were used to quantify cytokine and chemokines from TLR stimulated PBMC supernatants using multiplex arrays and plotted into floating bars with scatter plots. Statistical comparison between the groups was done with Mann-Whitney test, all *p*<0.05 indicated with asterisks * *p*<0.05-0.01 with **, *p*<0.01-0.001 with ***, and *p*<0.001.


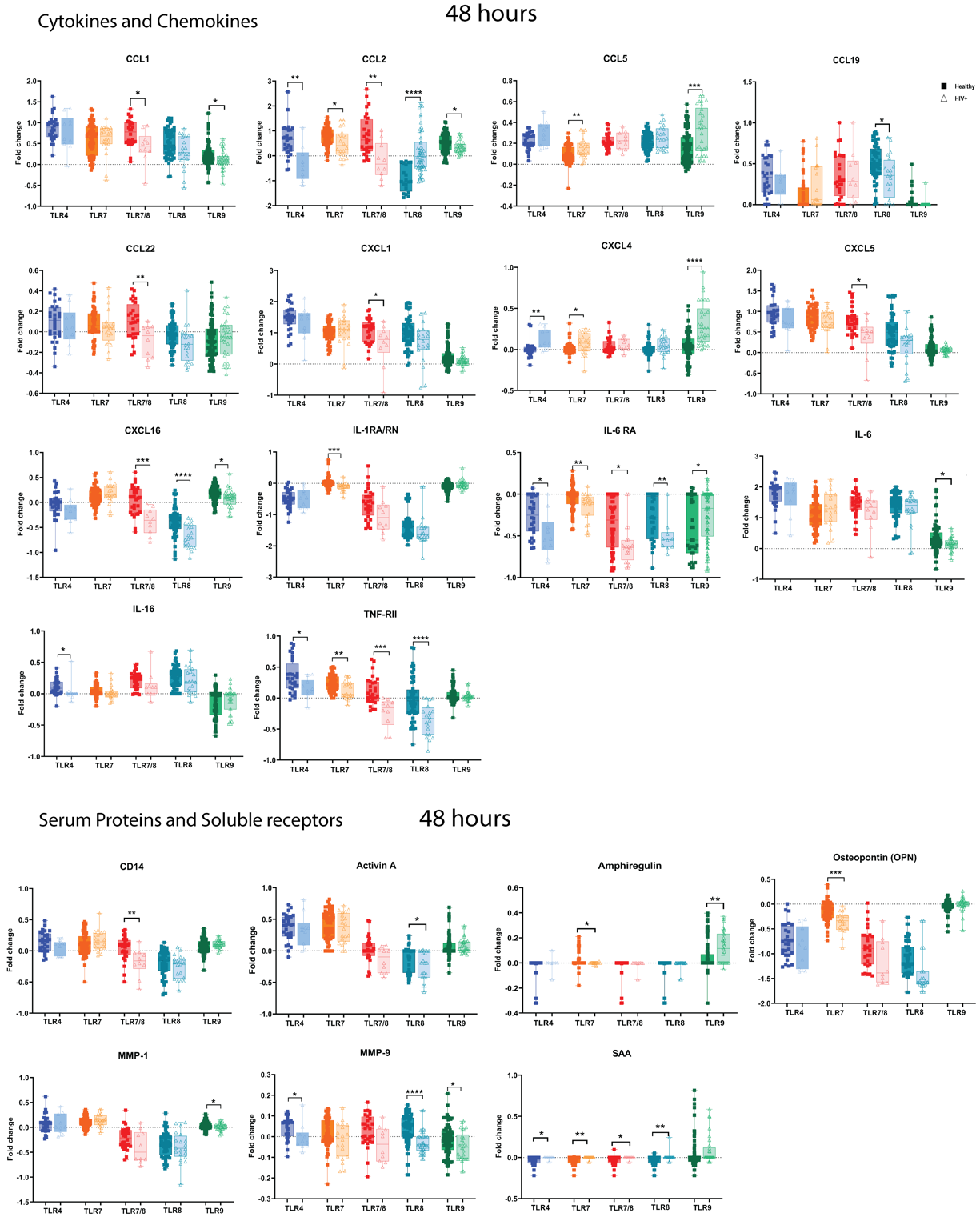


**Supplementary Figure 9.** Peripheral blood mononuclear cells were stimulated with TLR4 (*E. coli* LPS), TLR7 (combined Imiquimod and vesatolimod), TLR7/8 (resiquimod), TLR8 (combined selgantolimod, motolimod), TLR9 (combined CpG 2395 and CpG 2006). Machine learning algorithm was used to acquire cell features from confocal images. The features were cell size- small round cells (A) and large round cells (B)-, cell death- annexin V, (C), nuclear fragmentation (D), phagocytosis (E) cytoplasmic actin contraction (F), dispersal (G) and the presence (H) or absence of podosomes (I) PBMCs. Floating bars and scatter graphs are used to compare the cell feature data from healthy (n=30) and HIV-positive PBMCs, (n=10) with statistical comparison being done using Mann-Whitney test, and *p<*0.05 indicated using *, *p*<0.01 with **, *p*<0.001 with *** and *p<*0.0001.


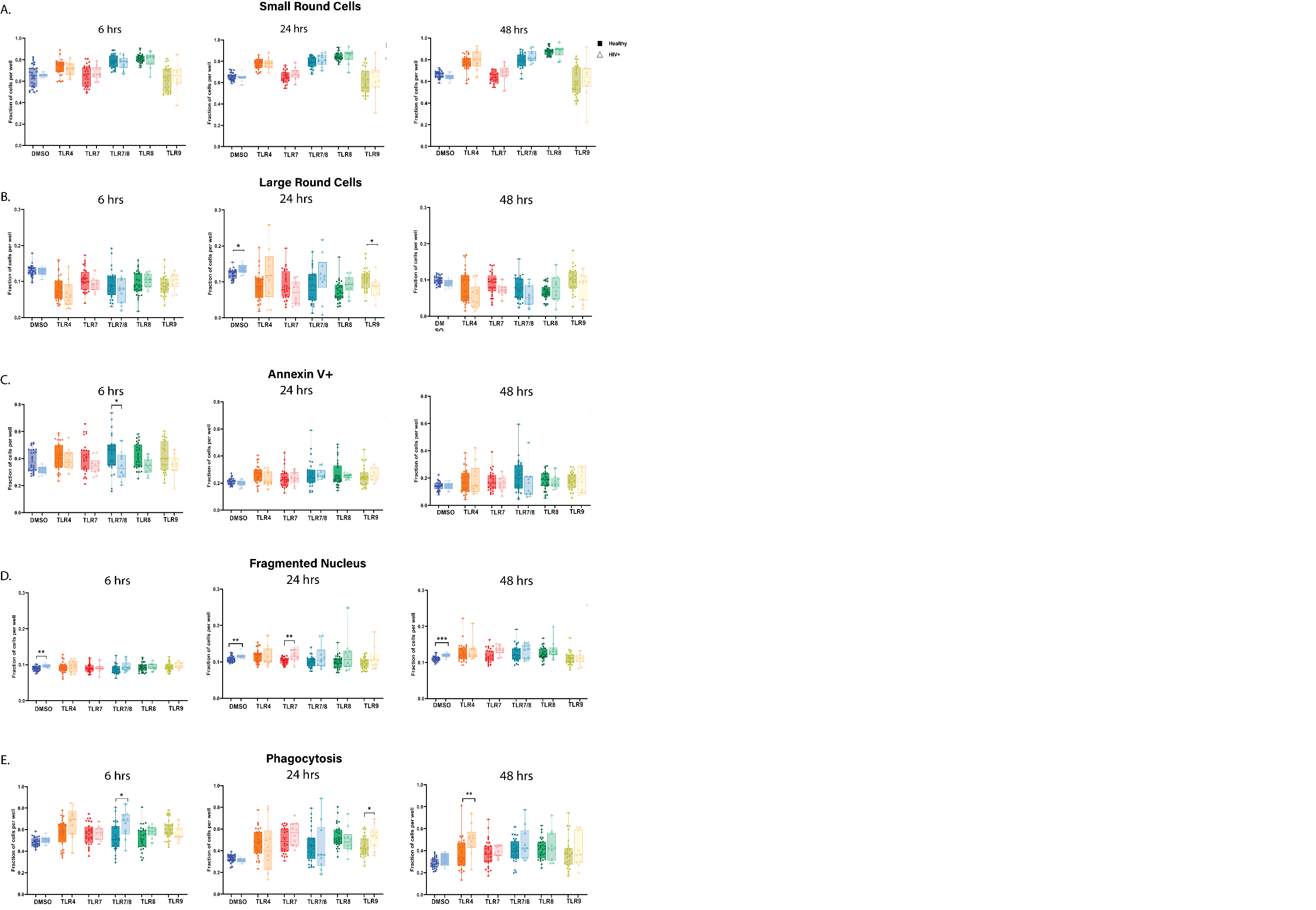


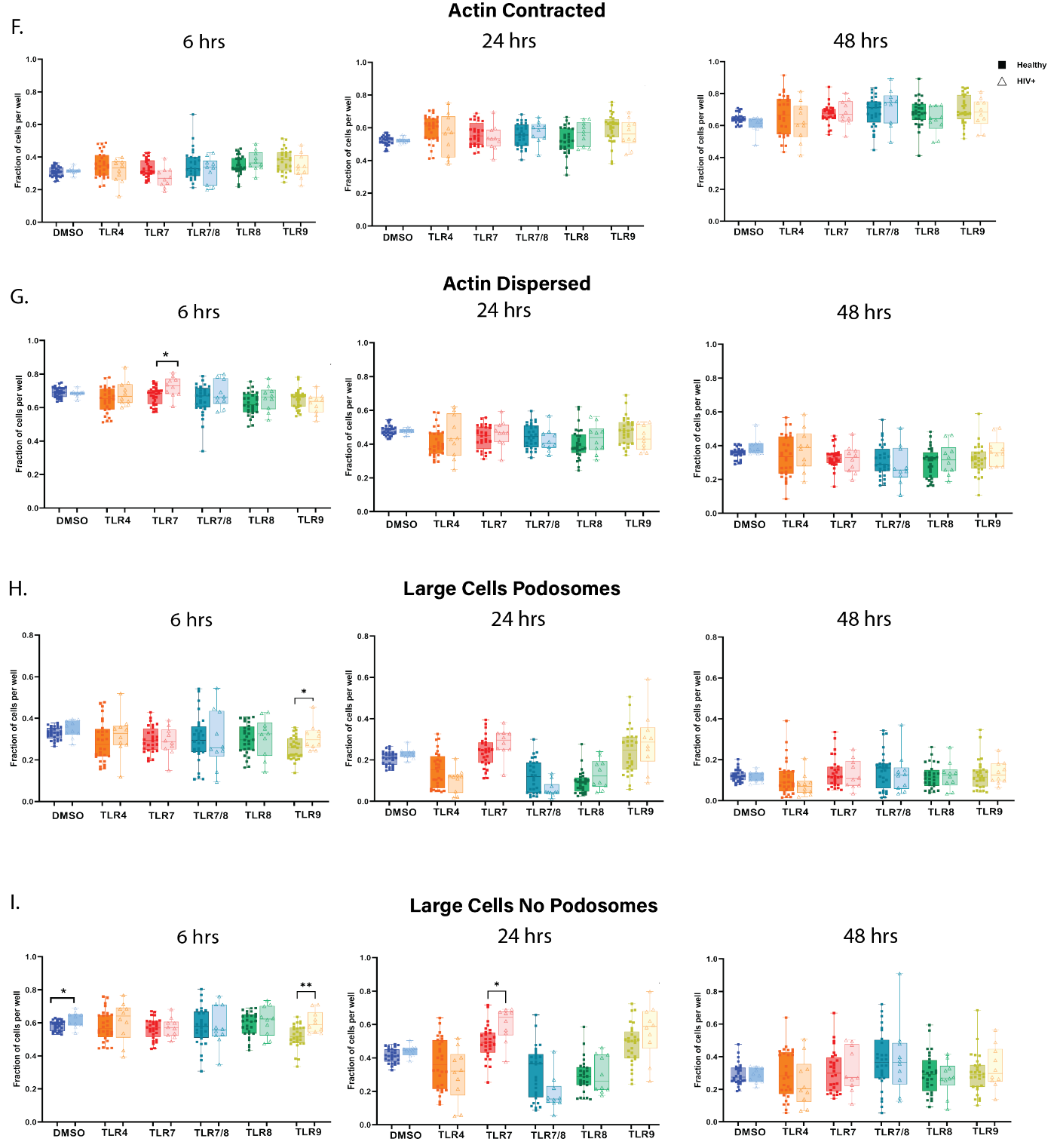


**References (Supplementary Materials)**

1. Li, S., et al. (2014). "Molecular signatures of antibody responses derived from a systems biology study of five human vaccines." Nat Immunol 15(2): 195-204.

2. Uhlen, M., et al. (2019). "A genome-wide transcriptomic analysis of protein-coding genes in human blood cells." Science 366(6472).
